# Site of vulnerability on SARS-CoV-2 spike induces broadly protective antibody against antigenically distinct Omicron subvariants

**DOI:** 10.1172/JCI166844

**Published:** 2023-04-17

**Authors:** Siriruk Changrob, Peter J. Halfmann, Hejun Liu, Jonathan L. Torres, Joshua J.C. McGrath, Gabriel Ozorowski, Lei Li, G. Dewey Wilbanks, Makoto Kuroda, Tadashi Maemura, Min Huang, Nai-Ying Zheng, Hannah L. Turner, Steven A. Erickson, Yanbin Fu, Atsuhiro Yasuhara, Gagandeep Singh, Brian Monahan, Jacob Mauldin, Komal Srivastava, Viviana Simon, Florian Krammer, D. Noah Sather, Andrew B. Ward, Ian A. Wilson, Yoshihiro Kawaoka, Patrick C. Wilson

**Affiliations:** 1 Drukier Institute for Children’s Health, Department of Pediatrics, Weill Cornell Medicine, New York, New York, USA.; 2 Influenza Research Institute, Department of Pathobiological Sciences, School of Veterinary Medicine, University of Wisconsin–Madison, Madison, Wisconsin, USA.; 3 Department of Integrative Structural and Computational Biology, The Scripps Research Institute, La Jolla, California, USA.; 4 University of Chicago Department of Medicine, Section of Rheumatology, Chicago, Illinois, USA.; 5 Department of Pathology, Molecular and Cell Based Medicine,; 6 Department of Microbiology,; 7 Center for Vaccine Research and Pandemic Preparedness,; 8 The Global Health and Emerging Pathogens Institute, and; 9 Division of Infectious Diseases, Department of Medicine, Icahn School of Medicine at Mount Sinai, New York, New York, USA.; 10 Center for Global Infectious Disease Research, Seattle Children’s Research Institute, Seattle, Washington, USA.; 11 Department of Pediatrics and; 12 Department of Global Health, University of Washington, Seattle, Washington, USA.; 13 The Skaggs Institute for Chemical Biology, The Scripps Research Institute, La Jolla, California, USA.; 14 Division of Virology, Department of Microbiology and Immunology, Institute of Medical Science, University of Tokyo, Tokyo, Japan.; 15 The Research Center for Global Viral Diseases, National Center for Global Health and Medicine Research Institute, Tokyo, Japan.; 16 Pandemic Preparedness, Infection and Advanced Research Center (UTOPIA), University of Tokyo, Tokyo, Japan.

**Keywords:** COVID-19, Immunology, Adaptive immunity, Immunoglobulins

## Abstract

The rapid evolution of the severe acute respiratory syndrome coronavirus 2 (SARS-CoV-2) Omicron variants has emphasized the need to identify antibodies with broad neutralizing capabilities to inform future monoclonal therapies and vaccination strategies. Herein, we identified S728-1157, a broadly neutralizing antibody (bnAb) targeting the receptor-binding site (RBS) that was derived from an individual previously infected with WT SARS-CoV-2 prior to the spread of variants of concern (VOCs). S728-1157 demonstrated broad cross-neutralization of all dominant variants, including D614G, Beta, Delta, Kappa, Mu, and Omicron (BA.1/BA.2/BA.2.75/BA.4/BA.5/BL.1/XBB). Furthermore, S728-1157 protected hamsters against in vivo challenges with WT, Delta, and BA.1 viruses. Structural analysis showed that this antibody targets a class 1/RBS-A epitope in the receptor binding domain via multiple hydrophobic and polar interactions with its heavy chain complementarity determining region 3 (CDR-H3), in addition to common motifs in CDR-H1/CDR-H2 of class 1/RBS-A antibodies. Importantly, this epitope was more readily accessible in the open and prefusion state, or in the hexaproline (6P)-stabilized spike constructs, as compared with diproline (2P) constructs. Overall, S728-1157 demonstrates broad therapeutic potential and may inform target-driven vaccine designs against future SARS-CoV-2 variants.

## Graphical abstract

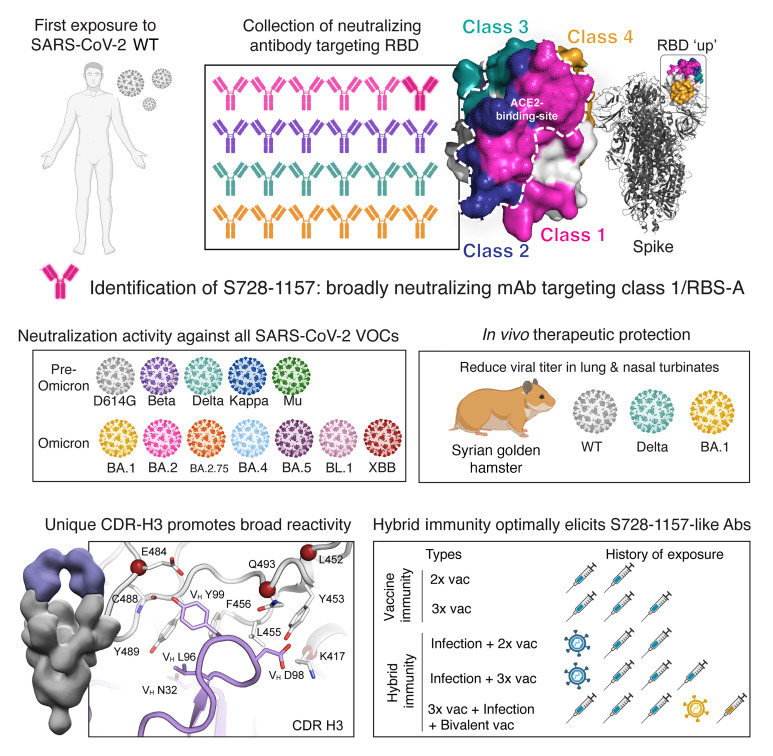

## Introduction

Since the start of the pandemic in December 2019, the severe acute respiratory syndrome coronavirus 2 (SARS-CoV-2) virus has led to over 660 million cases of coronavirus disease 2019 (COVID-19) and over 6.5 million deaths globally. Although the rapid development and distribution of vaccines and therapeutics have curbed the effect of COVID-19 to a large extent, the emergence of circulating variants of concern (VOCs) continues to represent a major threat due to the potential for further immune evasion and enhanced pathogenicity. The D614G variant was the earliest variant to emerge and became universally prevalent thereafter. Compared with WT, the D614G variant exhibited increased transmissibility rather than increased pathogenicity and was therefore unlikely to reduce efficacy of vaccines in clinical trials (1). Between the emergence of D614G to October of 2021, 4 additional significant VOCs evolved worldwide, including Alpha, Beta, Gamma, and Delta. Among these variants, Delta became a serious global threat because of its transmissibility, increased disease severity, and partial immune evasion, as shown by the reduced ability of polyclonal serum and mAbs to neutralize this strain (2–6). Shortly afterward, in November of 2021, the Omicron variant was identified and announced as a novel VOC. This variant possessed the largest number of mutations to date and appeared to spread more rapidly than previous strains (7, 8). Currently, there are a wide range of Omicron sublineages leading to new COVID-19 cases, with BQ.1, BQ.1.1 and XBB.1.5 becoming dominant over BA.5 and accounting for most new cases worldwide at the time of writing. The Omicron variants can escape recognition by COVID-19 vaccine-associated immunity to varying extents, thereby significantly reducing the neutralizing potency of serum antibodies from convalescent, fully mRNA-vaccinated individuals and individuals boosted with the new WT/BA.5 bivalent mRNA vaccine (9, 10). Similarly, Omicron variants were able to escape binding of several emergency use-authorization (EUA) therapeutic mAbs, even though these had been previously shown to be effective against earlier VOCs (10–12). Due to the lowered neutralization against Omicron and the continued threat of future VOCs, there is an urgent need to identify broad and potent neutralizing antibodies that can protect against diverse, evolving SARS-CoV-2 lineages.

In this study, we identified a potent receptor-binding domain–reactive (RBD-reactive) mAb from the peripheral blood of a SARS-CoV-2–convalescent individual that effectively neutralized Alpha, Beta, Kappa, Delta, Mu, and Omicron variants (BA.1, BA.2, BA.2.75, BA.4, BA.5, BL.1 and XBB). This mAb, S728-1157, significantly reduced BA.1 Omicron, Delta, and WT viral loads in the lungs and nasal mucosa following in vivo challenge in hamsters. S728-1157 binds the receptor binding site (RBS) that is fully exposed when the RBD on the spike is in the up conformation. The mAb uses motifs found in CDR-H1 and CDR-H2 that are common to IGHV3-53/3-66 class 1/RBS-A antibodies (13, 14), but also through extensive unique contacts with CDR-H3 to circumvent mutations in the VOCs spikes. This suggests that the rational design of future vaccine boosts covering Omicron variants should be modified to present stabilized spike in the mostly up configuration to optimally induce class 1/RBS-A mAbs that have similar CDR-H3 features.

## Results

### Isolation of RBD-reactive mAbs that exhibit diverse patterns of neutralization and potency.

**Figure 1:**
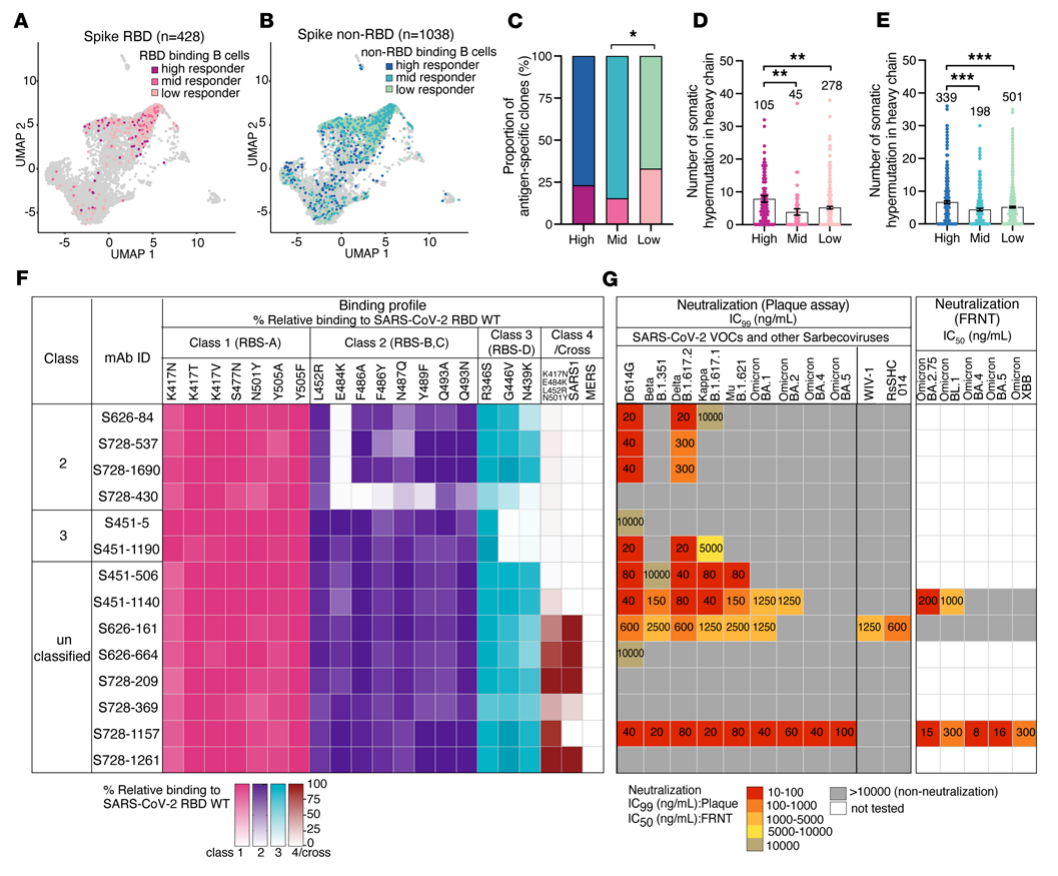
Characterization of RBD-reactive mAbs isolated from COVID-19–convalescent individuals. (**A** and **B**) Uniform manifold approximation and projection (UMAP) of SARS-CoV-2 (**A**) spike RBD binding and (**B**) spike non-RBD binding B cells isolated from convalescent individuals that could be characterized into 3 groups (high, mid, and low responders) based on their serological response against SARS-CoV-2 spike^13^. (**C**) Proportion of spike non-RBD- and spike RBD–specific binding B cells in each responder group. Colors in **A** and **B** are representative of antigen-specific B cells from each responder group. (**D**–**E**) Number of somatic hypermutations in the IGHV in antibodies targeting (**D**) RBD and (**E**) non-RBD. Data in **D**–**E** represent mean ± SEM. (**F**) Binding profile of RBD-reactive mAbs against RBD mutants associated with different antibody classes, a combinatorial RBD mutant, and the RBDs of SARS-CoV-1 and MERS-CoV. Color gradients indicate relative binding percentage compared with RBD WT. (**G**) Neutralization potency measured by plaque assay (complete inhibitory concentration; IC_99_) and FRNT. IC_50_, half inhibitory concentration of RBD-reactive mAbs to SARS-CoV-2 variants and sarbecoviruses. The statistical analysis in **C** was determined using Tukey multiple pairwise-comparisons and in **D** and **E** was determined using Kruskal-Wallis with Dunn’s multiple comparison test. Data in **F** and **G** are representative of 2 independent experiments performed in triplicate. Genetic information for each antibody is in Supplemental Table 3. The SARS-CoV-2 viruses used in the neutralization assay are indicated in Supplemental Table 5. **P ≤ 0.01; ***P ≤ 0.001.

Before the spread of the Omicron lineages, we previously characterized 43 mAbs targeting distinct epitopes on the spike protein, including the N-terminal domain (NTD), RBD, and subunit 2 (S2). None of these antibodies were able to neutralize the SARS-CoV-2 variants circulating at that time (15). In this study, an additional panel of RBD-reactive mAbs were expressed from 3 high-responder individuals who mounted robust anti-spike IgG responses, as defined previously (16) (Supplemental Tables 1 and 3; supplemental material available online with this article; https://doi.org/10.1172/JCI166844DS1). Although the proportion of spike RBD–binding B cells was similar in high-responders compared with mid- and low-responders (Figure 1, A–C), heavy chain somatic hypermutation rates were significantly greater in the high-responder group (Figure 1, D and E), suggesting that these individuals may have the highest potential to generate potent cross-reactive mAbs (16). These antibodies were further investigated against RBD mutants to identify their epitope classifications (17). Among 14 RBD-reactive mAbs, we identified 4 class 2 mAbs, 2 class 3 mAbs, and 8 unclassified mAbs that showed little to no reduction in binding against any key RBD mutants tested (Figure 1F). To be noted, class 2, class 3, and class 4 antibodies approximately correspond to the RBS B-D, S309, and CR3022 epitopes defined in previous studies (13, 18). Class 2 and 3 RBD mAbs did not recognize a multivariant RBD mutant containing K417N/E484K/L452R/N501Y substitutions, an artificially designed RBD to include key mutations for virus escape (17, 18), nor did they demonstrate any cross-reactivity to the RBD of SARS-CoV-1 and Middle Eastern respiratory syndrome (MERS)-CoV (Figure 1F). Functionally, class 2 and 3 RBD mAbs potently neutralized D614G and Delta variants, but neutralizing activity was more limited against Beta, Kappa and Mu (Figure 1G). None of the class 2 or 3 antibodies assayed neutralized any tested Omicron variant.

In contrast, the majority of unclassified mAbs bound to the RBD multivariant and cross-reacted to the SARS-CoV-1 RBD (Figure 1F). Among these, we identified 3 mAbs, S451-1140, S626-161, and S728-1157, which showed high neutralization potency against D614G and cross-neutralized Beta, Delta, Kappa, Mu, and Omicron BA.1 with 99% inhibitory concentration (IC_99_) in the range of 20–2,500 ng/mL (Figure 1G). Given the broad neutralization potency of these 3 mAbs, in addition to the plaque assay platform, we also performed the neutralization activity against authentic BA.2.75, BL.1 (BA.2.75+R346T), BA.4, BA.5, and XBB viruses using focus reduction neutralization test (FRNT) (Figure 1G). Of these, S728-1157 displayed high neutralizing activities against the panel of Omicron variants including BA.1, BA.2, BA.4, and BA.5, with an IC_99_ up to 100 ng/mL as measured by a plaque assay. A similar scenario was observed using FRNT, where S728-1157 maintained its high neutralization activity against BA.2.75, BL.1, BA.4, BA.5, and XBB with an IC_50_ in the range of 8–300 ng/mL (Figure 1G). S451-1140 neutralized BA.1, BA.2, BA.2.75, and BL.1 potently, but not BA.4 and BA.5, as observed in both neutralization assay platforms. On the other hand, S626-161 did not demonstrate neutralizing activity against Omicron variants beyond the BA.1 variant (Figure 1G). Although S626-161 had a lower neutralization potency against the tested VOCs than the other 2 antibodies, it was the only mAb that showed cross-reactivity not only to SARS-CoV-1 RBD but was also able to neutralize bat coronaviruses WIV-1 and RsSHC014 (Figure 1, F and G). These data suggest that S626-161 recognizes a conserved epitope that is shared between these sarbecovirus lineages but is absent in BA.2 and later strains. Additionally, compared with S728-1157 and S451-1140, S626-161 has a longer CDR-H3 that could provide an enhanced capability to recognize a highly conserved patch of residues shared across sarbecoviruses, as described in a previous study (19) (Supplemental Figure 1). When comparing immunoglobulin heavy (IGHV) and light chain (IGLV or IGKV) variable genes of these 3 mAbs with the available SARS-CoV-2 neutralizing mAbs database (13, 15, 20–27), we found that heavy chain variable genes utilized by S728-1157 (IGHV3-66), S451-1140 (IGHV3-23), and S626-161 (IGHV4-39) have been previously reported to encode several potently neutralizing SARS-CoV-2 antibodies targeting the RBD (21, 22, 28, 29). However, only S728-1157 had unique heavy and light chain variable gene pairings that have not been reported in the database (Supplemental Table 3), indicating that it is not a public clonotype.

**Figure 2:**
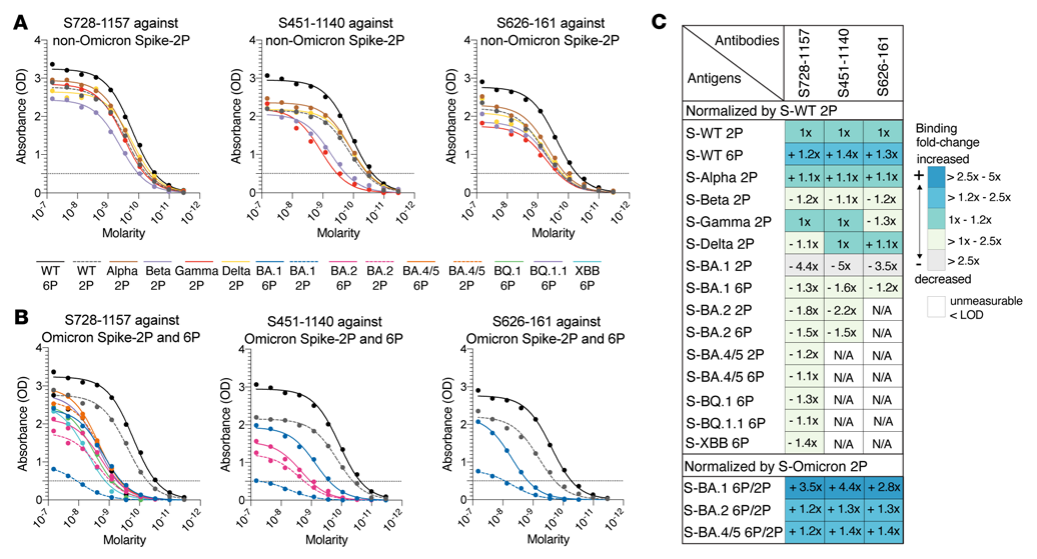
Binding breadth of Omicron-neutralizing mAbs. (**A** and **B**) Binding profile of S728-1157, S451-1140, and S626-161 against full-length spike SARS-CoV-2 variants determined by ELISA is shown for (**A**) non-Omicron variants and (**B**) Omicron sublineages. Dashed line in **A** and **B** indicate the limit of detection (LOD). (**C**) Heatmap represents AUC fold change of neutralizing RBD-reactive mAbs against ectodomain spike SARS-CoV-2 variants relative to WT-2P and the differences of AUC fold-change between spike Omicron-2P relative to spike Omicron-6P (BA.1, BA.2 and BA4/5). Data in **A** and **B** are representative of 3 independent experiments performed in triplicate. The full-length spike SARS-CoV-2 variants used in A and B are detailed in Supplemental Table 4.

These 3 mAbs (S451-1140, S626-161, and S728-1157) were characterized further to determine their binding breadth against SARS-CoV-2 VOCs (Figure 2, A and B). The prefusion-stabilized spike containing 2-proline substitutions in the S2 subunit (2P; diproline) has been shown to be a superior immunogen compared with the WT spike and is the basis of several current SARS-CoV-2 vaccines, including mRNA-based vaccines (30, 31). More recently, spike protein stabilized with 6 prolines (6P; hexaproline) was reported to boost expression and be even more stable than the original diproline construct; as a result, it has been proposed for use in the next generation of COVID-19 vaccines (32, 33). To determine whether there are antigenicity differences between the diproline and hexaproline spike constructs, both immunogens were included in our test panel. As measured by ELISA, we found that 3 mAbs bound 6P-WT spike antigen to a greater extent compared with WT-2P spike (Figure 2, A and B). All 3 mAbs showed comparable binding to the spikes of Alpha, Beta, Gamma, and Delta viruses, relative to that of WT-2P (Figure 2, A and B). However, the binding reactivities of these 3 mAbs were substantially reduced against a panel of Omicron-family antigens (Figure 2, B and C). S451-1140 binding was sensitive to mutations found in BA.1 and BA.2, resulting in a large decreased in binding and a 31-fold decrease in neutralization against these variants compared with WT-2P antigen and D614G virus, respectively (Figure 2B). The sarbecovirus-cross neutralizing mAb, S626-161, also showed 1.2- to 3.5-fold reduced binding to spike BA.1 antigens, which may account for a 2-fold reduction in neutralization activity against BA.1 (Figure 1G and Figure 2, B and C). For the most potent broadly neutralizing antibody (bnAb), S728-1157, binding to Omicron antigens was reduced to a lesser extent (ranging from 1.1- to 4.4-fold) compared with WT-2P spike and was unaffected in neutralizing activity (Figure 1G and Figure 2, B and C). The substantial decrease in the Omicron-neutralizing mAb binding to the BA.1 spike may be due to alterations in its mobility and related to the tight packing of the Omicron 3-RBD-down structures and preference for 1-up RBD that aid in evading antibodies, as reported by a previous study (34). The 2P and 6P stabilizing mutations also have differential effects in Omicron variants where all 3 mAbs showed over 2.8-fold increased binding to spike BA.1-6P compared with the BA.1-2P version, but only marginally increased binding to spike BA.2 and BA.4/5 6P versions compared with their 2P versions by 1.2 × to 1.4 ×, suggesting slightly better accessibility of Omicron-neutralizing mAbs to the hexaproline versions, especially for the spike BA.1 construct. In addition to ELISA, biolayer interferometry (BLI) was used to quantify the binding rate and equilibrium constants (k_on_, k_off_, and K_D_) of these 3 mAbs to a panel of spike antigens (Supplemental Figure 2). The recognition k_on_ rates of the fragment antigen binding (Fab) to hexaproline spikes were 1.5- to 3.3-fold faster when compared with diproline spikes (Supplemental Figure 2, B and C), showing that the antibodies bound to the 6P construct more rapidly than to the 2P construct. This might have been expected if the epitopes were more accessible on the RBD in the open state on the hexaproline spike. Except for S626-161, Fabs dissociated from the hexaproline spike more slowly (had a lower k_off_) than the diproline spike, such that the overall K_D_ showed that S728-1157 and S451-1140 bound to the hexaproline spike with greater affinity (Supplemental Figure 2, B and C). The increase in binding to the hexaproline spike was even more notable for intact IgG by the 1:2 interaction model as shown by S728-1157 and S451-1140 mAbs, consistent with exposure of multiple epitopes with 6P stabilization allowing improved avidity (Supplemental Figure 2, A and C). Taken together, these results suggest that the epitopes targeted may be comparatively more accessible on the 6P-stabilized spike when the RBD is in the open state. Structural analyses were next performed to verify this conjecture.

### Structural analysis of broadly neutralizing mAbs.

**Figure 3:**
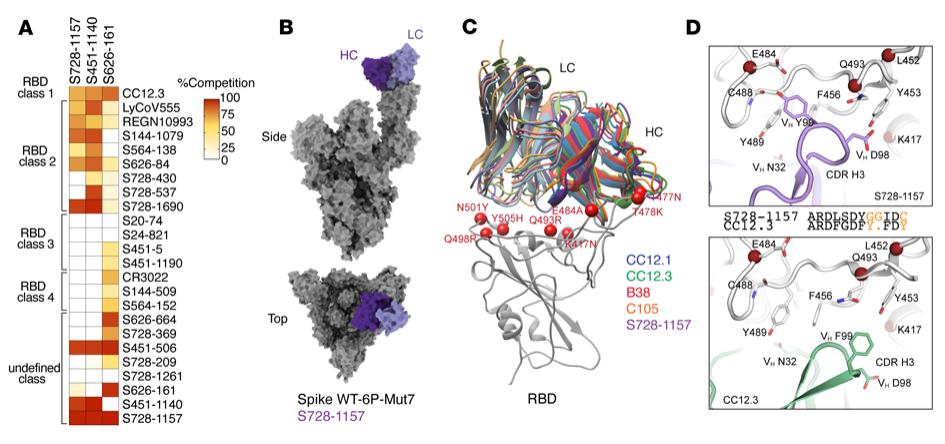
Mechanism of broad neutralization of S728-1157. (**A**) Epitope binning of broadly neutralizing RBD-reactive mAbs. Heatmap demonstrating the percentage of competition between each RBD-reactive mAb from previous studies (15, 23, 36-38) with 3 broadly neutralizing mAbs, S728-1157, S451-1140, and S626-161. Data are representative of 2 independent experiments performed in triplicate. (**B**) Surface representation of the model derived from the cryoEM map of spike WT-6P-Mut7 in complex with IgG S728-1157. The heavy chain is shown in dark purple, light chain in light purple, and the spike protein in gray. Although we observe full mAb occupancy in the cryo-EM map, only 1 Fv is shown here. (**C**) Structural comparison of S728-1157 to other RBS-A/class 1 antibodies such as CC12.1 (PDB ID: 6XC2, blue), CC12.3 (PDB ID: 6XC4, green), B38 (PDB ID: 7BZ5, red), and C105 (PDB ID: 6XCN, orange). The heavy chains are in a darker shade, and the light chains in a lighter shade of their respective colors. Omicron BA.1 mutations near the epitope interface are shown as red spheres. (**D**) CDR-H3 forms distinct interactions with SARS-CoV-2 RBD between S728-1157 and CC12.3. Sequence alignment of CDR-H3 of the 2 antibodies are shown in the middle with nonconserved residues shown in orange.

As a first approximation of epitopes bound, an ELISA competition assay was used to determine whether these 3 broadly neutralizing mAbs shared any overlap with our current panel of mAbs, a collection of mAbs with known epitope specificities from previous studies (15, 25, 35), and 2 other mAbs currently in clinical use, LY-CoV555 (Eli Lilly) (36) and REGN10933 (Regeneron) (37). The binding sites of S451-1140 and S728-1157 partially overlapped with CC12.3 (23, 25), a class 1 neutralizing antibody, and most class 2 antibodies, including LY-CoV555 and REGN10933, but not with class 3 and class 4 antibodies (Figure 3A). S626-161 shared a notable overlap in binding region with class 1 CC12.3, several class 4 antibodies, including CR3022, and other unclassified antibodies, while having some partial overlap with several class 2 and a single class 3 antibody (Figure 3A). Analogously, a competition BLI assay revealed that S451-1140 and S728-1157 strongly competed with one another for binding to spike WT-6P, whereas S626-161 did not (Supplemental Figure 3). Overall, these data suggest S451-1140 and S728-1157 recognize similar epitopes that are distinct from S626-161.

Antibody S728-1157 was encoded by IGHV3-66 and possessed a short complementarity determining region 3 (CDR-H3). Notably, mAbs that bind the RBS in binding mode 1 (i.e. RBS-A or class 1 site), typified by CC12.1, CC12.3, B38, and C105 (13, 18, 23, 29, 38, 39), tend to use IGHV3-53 or 3-66 and are sensitive to VOC mutations (40). However, the CDR-H3 region of S728-1157 is highly distinct from other antibodies of this class, potentially accounting for its broader activity. To understand the structural basis of broad neutralization by S728-1157 at this epitope, we resolved a cryo-electron microscopy (cryo-EM) structure (Figure 3B) of IgG S728-1157 in complex with spike WT-6P-Mut7, a version of spike WT-6P possessing an interprotomer disulfide bond at C705 and C883, at approximately3.3 Å global resolution (Supplemental Figure 4E). Using symmetry expansion, focused classification, and refinement methods, we achieved local resolution at the RBD-Fv interface to approximately 4 Å (Supplemental Figure 4E and Supplemental Table 8). A crystal structure of S728-1157 Fab was determined at 3.1 Å resolution and used to build the atomic model at the RBD-Fv interface. Our structures confirm that S728-1157 bound the RBS-A (or class 1) epitope in the RBD-up conformation (Figure 3B and Supplemental Figure 4E), similar to other IGHV3-53/3-66 antibodies (Figure 3C). Steric blockage of the angiotensin converting enzyme 2 (ACE2) binding site by S728-1157 explains its high neutralization potency against SARS-CoV-2. The _32_NY_33_ motif and _53_SGGS_56_ motif (23) in S728-1157 CDR-H1 and -H2 interact with the RBD in almost the same way as CC12.3 (Supplemental Figure 4, B and C). However, compared with V_H__98_DF_99_ in CC12.3, V_H__98_DY_99_ in S728-1157 CDR-H3 forms more extensive interactions, including both hydrophobic and polar interactions, with the RBD, which may account for the broad neutralization against VOCs (Figure 3D and Supplemental Tables 6 and 7). The diglycine V_H__100_GG_101_ in S728-1157 CDR-H3 may also facilitate more extensive binding compared with V_H_ Y_100_ in CC12.3, likely due to the flexibility in the glycine residues that lead to a different conformation of the tip of the CDR-H3 loop and a relative shift of residues at _98_DY_99_.

Although the Omicron VOCs have extensive mutations in the RBD, most of these residues do not interact with S728-1157, as binding is still observed (Supplemental Figure 4A). From our spike WT-6P-Mut7 + Fab S728-1157 model, Y505 to V_L_ Q31, and E484 to V_H_ Y99 are predicted to make hydrogen bonds (Supplemental Figure 4D and Supplemental Table 6), which have the potential to be disrupted by Omicron mutations Y505H and E484A. However, a Y505H mutation would still allow for a hydrogen bond with V_L_ Q31, and an E484A mutation would add another hydrophobic side chain near hydrophobic residues V_L_ Y99, F456, and Y489. These contacts may explain, in part, the mechanism that enables S728-1157 to retain neutralizing activity, albeit reduced, against the spike BA.1 antigen (Figure 1G and Figure 2B). The BA.1 antigen, in turn, is possibly related to the Omicron mutations altering the conformational landscape of the spike protein (34). However, several somatically mutated residues, i.e., V_H_ L27, L28, R31, F58, and V_L_ V28 and Q31, in S728-1157 are involved in interaction with SARS-CoV-2 RBD (Supplemental Figure 1 and Supplemental Table 7), which may also contribute to its broad reactivity compared with CC12.3. Overall, our structural studies revealed the basis of broad neutralization of S728-1157 that can accommodate most mutations in the SARS-CoV-2 VOCs.

### S728-1157 reduces replication of SARS-CoV-2 BA.1 Omicron, Delta, and WT SARS-CoV-2 in Syrian hamsters.

**Figure 4:**
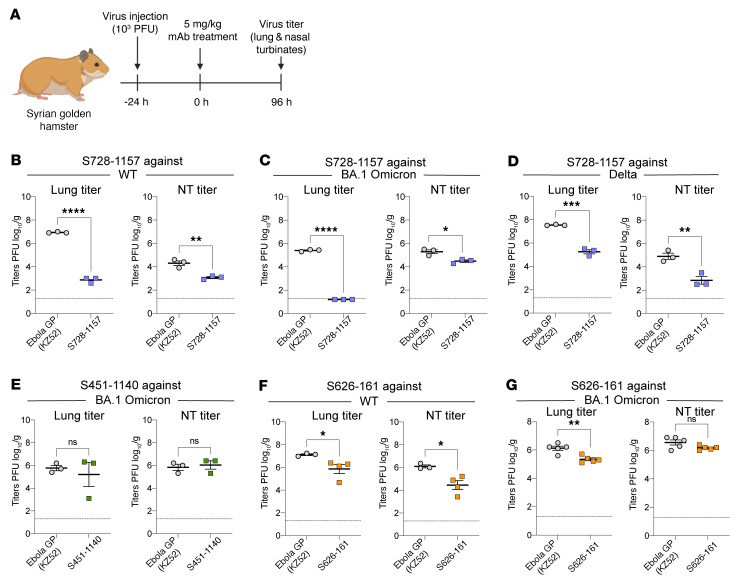
Protective efficacy of bnAbs against SARS-CoV-2 infection in hamsters. (**A**) Schematic illustrating the in vivo experiment schedule. Lung and nasal turbinate (NT) viral replication SARS-CoV-2 are shown for hamsters treated therapeutically with (**B**–**D**) S728-1157 (*n* = 3) (**E**) S451-1140 (*n* = 3) and (**F** and **G**) S626-161 (*n* = 4) at day 4 after challenge with SARS-CoV-2 compared with a control mAb, anti-Ebola surface glycoprotein (KZ52) antibody. Dashed horizontal lines represent the limit of detection (LOD) of the experiment. *P* values in (**B**–**G**) were calculated using Unpaired 2-tailed *t* test. The SARS-CoV-2 viruses used for infection are detailed in Supplemental Table 5. *P ≤ 0.05; **P ≤ 0.01; ***P ≤ 0.001; ****P < 0.0001.

To evaluate the protective efficacy of our broadly neutralizing mAbs, we utilized a golden Syrian hamster infection model that has been widely used for SARS-CoV-2. Hamsters received 5 mg/kg of our test mAbs or an isotype control targeting an irrelevant antigen (ebolavirus glycoprotein) via intraperitoneal injection 1 day after infection with SARS-CoV-2 viruses. Lung and nasal tissues were collected 4 days after infection (Figure 4A). Therapeutic administration of S728-1157 resulted in reduced titers of WT, BA.1 Omicron, and Delta variants in both the nasal turbinates and lungs of infected hamsters (Figure 4, B–D). Interestingly, the effect of S728-1157 in the lungs was dramatic, reducing WT and BA.1 Omicron viral loads by approximately 10^4^ PFU, with the viral titers of the BA.1 Omicron variant being completely abolished (Figure 4C). In contrast to in vitro neutralization (Figure 1G), S451-1140 did not reduce BA.1 Omicron viral replication in lung and nasal turbinates, indicating a disconnect between in vitro neutralization and in vivo protection for this clone (Figure 4E). In comparison, S626-161 administration resulted in marginally significant reductions in lung viral titers following WT and BA.1 challenge (Figure 4, F and G). These data underscore that, to precisely define broadly protective mAbs, evaluating protection efficacy in parallel with neutralization activity is required. Moving forward, it will be interesting to examine to what extent the protective capacity of S728-1157 is Fc-dependent. Overall, S728-1157 represents a promising mAb with broad neutralization efficacy against SARS-CoV-2 variants that is capable of dramatically reducing WT, Delta, and BA.1 replication in vivo.

### SARS-CoV-2 infection rarely elicits potent S728-1157–like cross-neutralizing mAbs.

**Figure 5:**
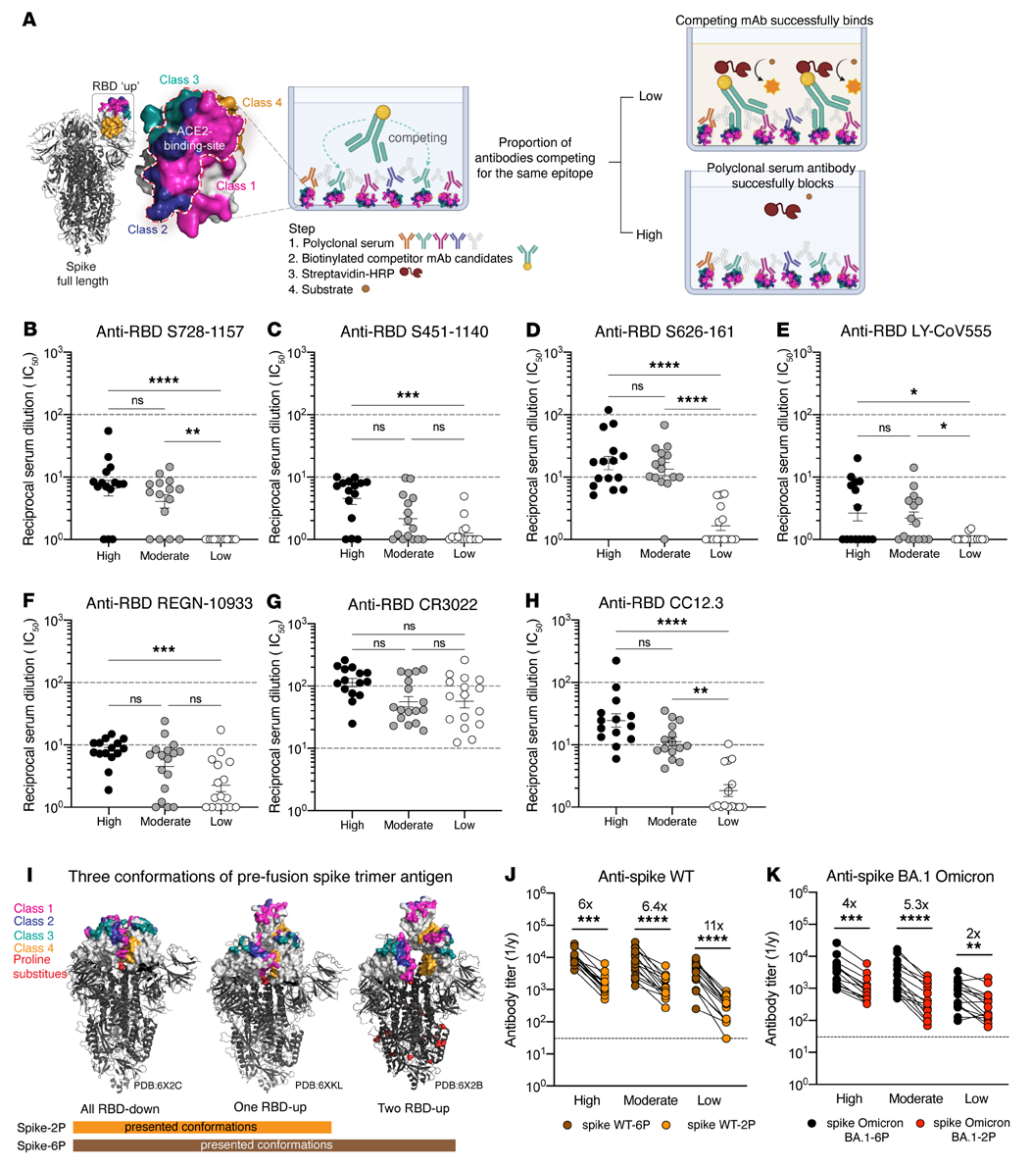
Convalescent serum antibody competition with broadly neutralizing RBD-reactive mAbs and comparison of serum antibody response against 6P- versus 2P-stabilized spikes. Schematic diagram for experimental procedure of serum competitive ELISA (**A**). The model created with BioRender.com. IC_50_ of polyclonal antibody serum from convalescent individuals (high responder, *n* = 15 donors; moderate responder, *n* = 16 donors; low responder, *n* = 16 donors) that could compete with broadly neutralizing mAbs (competitor mAb) S728-1157 (**B**), S451-1140 (**C**), and S626-161 (**D**), as well as therapeutic mAbs LY-CoV555 (**E**), REGN-10933 (**F**), nonneutralizing mAb CR3022 (**G**), and well-defined RBS-A/class 1 mAb CC12.3 (**H**). The reciprocal serum dilutions in **B**–**H** are showed as Log1P of the IC_50_ of serum dilution that can achieve 50% competition with the competitor mAb of interest. The statistical analysis in **B**–**H** was determined using Kruskal-Wallis with Dunn’s multiple comparison test. Representative 3 conformations of prefusion spike trimer antigen observed in the previous structural characterization of SARS-CoV-2 stabilized by 2P and 6P (33, 49) (**I**). Endpoint titer of convalescent sera against SARS-CoV-2 spike WT (**J**) and Omicron BA.1 (**K**) in 2 versions of spike substituted by 2P and 6P. Data in **B**–**H** and **J**–**K** are representative of 2 independent experiments performed in duplicate. Wilcoxon matched-pairs signed rank test was used to compare the anti-spike antibody titer against 2P and 6P in **J** and **K**. Fold change indicated in **J** and **K** is defined as the mean fold change.*P ≤ 0.05; **P ≤ 0.01; ***P ≤ 0.001; ****P < 0.0001. Biorender.com was used to create panel A.

Given the cross-neutralization and prophylactic potential of S728-1157, we sought to evaluate whether S728-1157–like antibodies are commonly induced among polyclonal responses in SARS-CoV-2 patients. To assess this, we performed competition ELISAs using convalescent serum to detect anti-RBD antibody titers that could compete for binding with S728-1157 (Figure 5A). Subjects were divided into 3 groups based on their magnitude of antibody responses, as defined previously (15, 16). Although high and moderate responders had higher titers of S728-1157–competitive serum antibodies compared with low responders (Figure 5B), the titers were quite low across all groups, suggesting that it is uncommon to acquire high levels of S728-1157–like antibodies in polyclonal serum following WT SARS-CoV-2 infection. In addition to S728-1157, we tested the competition of convalescent serum with other mAbs, including S451-1140, S626-161, LY-CoV555, REGN10933, CR3022, and CC12.3. Similar to S728-1157, we observed relatively low titers of antibodies competing with S451-1140, S626-161, LY-CoV555, REGN10933, and CC12.3 in polyclonal serum from most of the convalescent individuals (Figure 5, C–F and H). Nonetheless, high responders tended to have significantly higher titers against those neutralizing mAbs than low responders (Figure 5, B–F and H). In contrast, antibodies targeting the CR3022 epitope site were more pronounced in convalescent individuals, suggesting the enrichment of class 4 RBD antibodies in polyclonal serum (Figure 5G). Notably, there was no significant difference in titers of CR3022 across the 3 responder groups, suggesting that CR3022-site antibodies were consistently induced during WT SARS-CoV-2 infection in most individuals. Interestingly, compared with CC12.3, S728-1157 was detected at 4-fold lower levels in the serum of high responders. Thus, despite class 1 antibodies being frequently induced by natural infection and vaccination (14, 20, 28, 29, 41–43), our data suggest that S728-1157–like antibodies that represent a subset of this class are comparatively rare.

Additionally, we examined the difference in reactivity to 2P- versus 6P-stabilized spike in our convalescent cohort sera (Figure 5, I–K). We found that all 3 responder groups mounted anti-spike reactive antibodies against 6P-stabilized spike WT to a greater extent than 2P-stabilized spike WT, by a factor of 6-to-11–fold (Figure 5J), indicating that the major antigenic epitopes were better exhibited or stabilized on 6P-stablized antigen. Using the same samples, high and moderate responders also had lower titers of anti-spike antibodies against BA.1-2P than BA.1-6P, by 4-to-5–fold (Figure 5K). Of note, low responders had a smaller fold change in binding reactivity against spike BA.1 Omicron-2P and 6P (2-fold reduction) compared with WT-2P and 6P spike (11-fold reduction) (Figure 5, J and K), suggesting that serum antibody against BA.1 Omicron-reactive epitopes may be more limited in low responder subjects. Overall, these data suggest that there is improved polyclonal binding induced by natural infection to 6P-stabilized spike, both for WT and Omicron viruses.

### S728-1157–like antibodies are optimally induced in the context of hybrid immunity.

**Figure 6:**
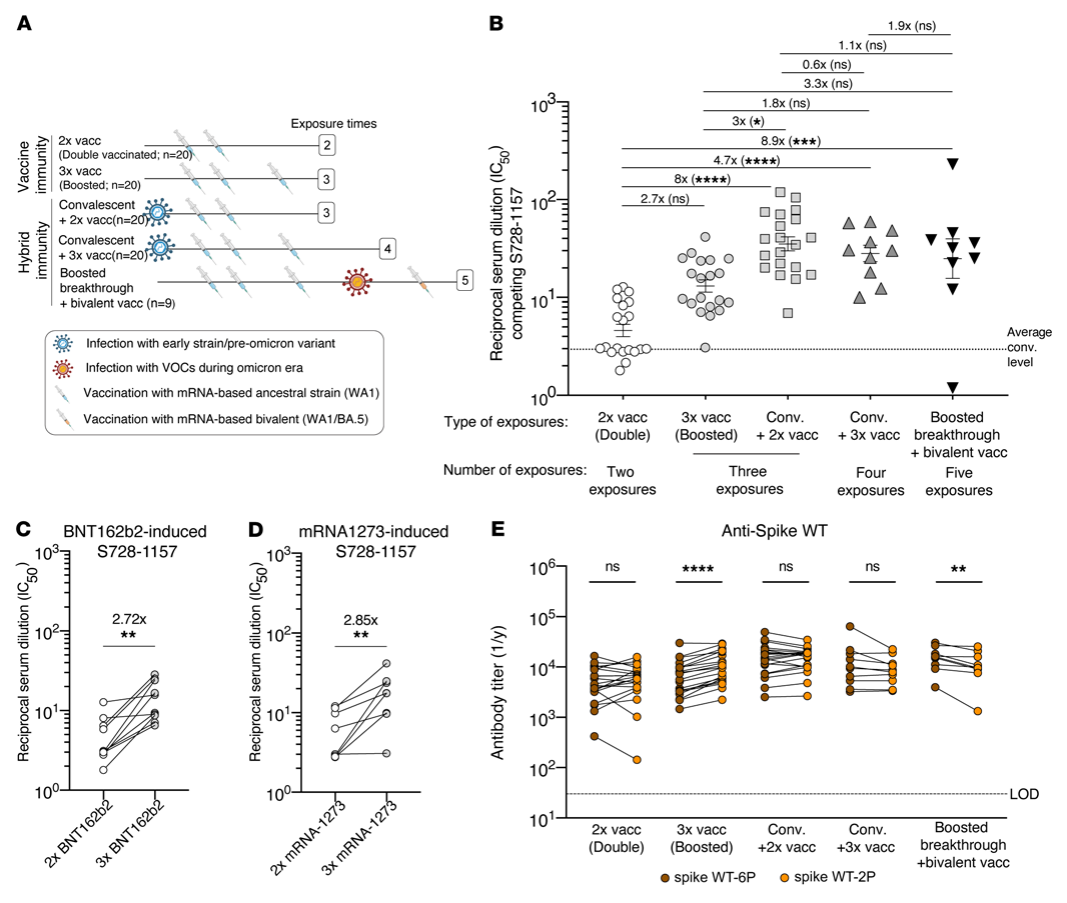
mRNA-vaccinated serum antibody competition with S728-1157 neutralizing RBD-reactive mAbs and comparison of serum antibody response against 6P- versus 2P-stabilized spikes. Collection of sera and exposure history from vaccine groups (**A**). 2 × vacc, double vaccination (WA-1), (*n* = 20 participants); 3 × vacc., boosted or triple vaccination (WA-1) (*n* = 20 participants); conv. + 2 × vacc., convalescent plus double vaccination (WA-1) (*n* = 20 participants); conv. + 3 × vacc., convalescent plus boosted/triple vaccination (WA-1) (*n* = 10 participants); boosted breakthrough + bivalent vacc., after-boost infection followed by bivalent vaccination (WA-1/BA.5) (*n* = 9 participants). The model created with BioRender.com. Fold change of IC_50_ of antibody competing for binding to the S728-1157 epitope from 5 groups of individuals who received mRNA-based vaccine with variety type of exposure history (**B**). Dashed line in B indicates average of antibody titer that was found in convalescent individuals related to Figure 4. The statistical analysis in B was determined using Kruskal-Wallis with Dunn’s multiple comparison test. Comparison of the kinetics of serum antibodies to the S728-1157 epitope present in a given participant after completion of the primary vaccination regimen (2 × vacc.) and after boosted vaccination (3 × vacc.) divided by vaccine types (**C** and **D**). The connecting lines in **C** and **D** identify paired samples. Endpoint titer of mRNA-based vaccinated sera against SARS-CoV-2 spike WT substituted by 2P and 6P (**E**). Dashed line in E indicates limit of detection (LOD) of the analysis. Wilcoxon matched-pairs signed rank test was used to compare the antibody titer in **C**–**E**. Fold change indicated in **B**–**D** is defined as the mean fold change. Data in **B**–**E** are representative of 2 independent experiments performed in duplicate. *P ≤ 0.05; **P ≤ 0.01; ***P ≤ 0.001; ****P < 0.0001. Biorender.com was used to create panel A.

Primary SARS-CoV-2 infection without vaccination has become rare in the current global setting, and several studies have reported that SARS-CoV-2 immunity differs between individuals with specific vaccination/infection histories. As a result, we next sought to investigate which common exposures, aside from WT infection with ancestral SARS-CoV-2 alone, would effectively induce S728-1157–like antibodies in plasma from monovalent mRNA-based vaccinees with and without prior infection. We obtained the necessary biospecimen from the Protection Associated with Rapid Immunity to SARS-CoV-2 (PARIS) study cohort, which has followed healthcare workers longitudinally since the beginning of the pandemic (44). We selected plasma samples from fully immunized (2 × vaccinated) study participants with and without infection as well as from boosted participants (3 × vaccinated) with and without infection. In addition, we also included samples from study participants who had received the bivalent mRNA vaccine (ancestral WA1/2020 plus Omicron BA.5) (Figure 6A and Supplemental Table 2). The breakthrough infections in participants who had received booster vaccinations occurred at a time when the Omicron lineages had displaced all other SARS-CoV-2 lineages in the New York metropolitan area. We found that double-vaccinated individuals had lowest titers of S728-1157 competitive serum antibodies among the 5 groups of samples tested (Figure 6B). Notably, these levels were similar to that observed for our convalescent-unvaccinated cohort (all responders; Figure 5B). In comparison, individuals with a history of natural infection, including convalescent individuals with 2 of 3 vaccine doses, and individuals that had experienced a breakthrough infection and received a bivalent booster, showed significantly higher levels of S728-1157 elicitation compared with uninfected but vaccinated individuals (Figure 6B). Although the uninfected 3-dose group displayed only a nonsignificant increase compared with the 2-dose group, paired breakdown by vaccine type indicated that homologous third doses of BNT162b2 and mRNA-1273 significantly increased S728-1157-like neutralizing antibody titers by 2.72 × and 2.85 ×, respectively (Figure 6, C and D). To note, among the participants with 3 total contacts with spike by any means, S728-1157-like antibody titers were 3 × higher in convalescent double-vaccinees compared with infection-naive triple-vaccinees, suggesting that SARS-CoV-2 infection more optimally induces this clonotype. Among hybrid immunity groups, we noted that a majority of the boosted individuals with breakthrough who received the bivalent booster vaccine dose had only marginally higher S728-1157 antibody titer compared with preomicron convalescent vaccinated groups, suggesting that the S728-1157 titer was likely approaching a plateau after 3 exposures. We also investigated the titers of polyclonal antibodies that competed with CC12.3 and CR3022 in addition to S728-1157. All individuals exhibited relatively high titers of CC12.3- and CR3022-like antibodies, independent of the number and type of exposures (Supplemental Figure 5), contrary to what we observed for S728-1157-like antibodies. Overall, these data indicate that SARS-CoV-2 infection and mRNA vaccination both contribute to S728-1157-like antibody induction, with infection playing a more dominant role in vaccinated individuals.

Finally, in comparing responses against 2P- versus 6P-stabilized spike in the mRNA-vaccination cohort, we found that most groups elicited similar levels of antibodies against both constructs. The exception to this was the uninfected triple-vaccinated group, who demonstrated statistically higher, though only slightly increased, reactivity to the 2P compared with the 6P-stabilized spike (Figure 6E). These data suggest that, in contrast to natural infection (Figure 5, J and K), vaccination alone produces a polyclonal response that is more restricted to epitopes in the Spike-2P construct, in line with the Spike-2P formulation of current vaccines. Ultimately, these findings support the idea that 6P-stabilization of future SARS-CoV-2 vaccines could be of major benefit in inducing broadly protective antibody clonotypes like S728-1157.

## Discussion

In this study, we identify a potent bnAb isolated from a memory B cell of an individual who had recovered from SARS-CoV-2 infection during the initial wave of the COVID-19 pandemic. This bnAb, S728-1157, maintained substantial binding reactivity and had consistent neutralizing activity against all tested SARS-CoV-2 VOC, including Omicron BA.1, BA.2, BA.2.75, BL.1 (BA.2.75+R346T), BA.4, BA.5, and XBB, and was able to substantially reduce infectious viral titers following Delta and BA.1 infection in hamsters.

We found that convalescent serum from our cohort contained low concentrations of antibodies that compete with S728-1157 (a class 1/ RBS-A antibody) and class 2 epitope mAbs. This suggests that S728-1157 is somewhat unique from other antibodies that target class 1 epitopes and is infrequently induced in the RBD-specific memory B cell pool. Instead, our natural infection cohort appeared to preferably induce antibodies targeting the CR3022 (class 4) epitope; antibodies of this specificity are often cross-reactive but less potently neutralizing than RBS-targeting antibodies (14, 17). These data are complementary to our previous findings demonstrating that an abundance of class 3/S309 antibodies in convalescent sera may contribute to neutralizing activity against Alpha and Gamma variants, whereas a lack of class 2 antibodies may account for reduced neutralization capability against Delta (15). Notwithstanding, the breadth of activity of most of these RBS-targeting antibodies (RBS-A/class 1, RBS-B,C/class 2 and RBS-D, S309/class 3) against Omicron variants is reported to be highly limited (11, 40, 45).

The key challenge moving forward will to be determine how to improve the elicitation of broadly cross–reactive antibodies to conserved RBS epitopes. In this regard, we observed here that individuals with hybrid immunity mounted significantly higher titers of S728-1157-like antibodies than vaccinated individuals without prior infection. Importantly, this phenomenon was noted even when the number of exposures was controlled for (i.e., in convalescent double vaccinees versus uninfected triple vaccinees), suggesting that some element of infection-associated immunity (or a vaccine formulation that can mimic this type of immunity) is important for the elicitation of this clonotype. This is consistent with experimental evidence documenting that individuals with hybrid immunity have broader antibody-reactivity profiles compared with those that only have vaccination-induced or primary infection–induced immune responses (9).

The structures herein illustrated that S728-1157 bound the RBS-A/class 1 epitope in the up conformation RBD. This epitope appears to be more readily accessible on 6P-stabilized spikes, which have been reported to present 2 RBDs in the up state, compared with 2P spikes, which present only 1 (30, 33, 46, 47), and to which our antibodies specific for up-conformation spike show improved binding. S728-1157 was isolated after natural infection; in such contexts, the odds of inducing S728-1157–like clones are likely higher given that the RBD must be able to adopt an up conformation, even transiently, to bind to ACE2, thereby exposing this epitope. Unlike the majority of IGHV3-53/3-66 RBS-A/class 1 antibodies, S728-1157 can accommodate key mutations in VOC spikes using extensive interactions between CDR-H3 and the RBD (29, 48–50). S728-1157 also uses a different light chain (IGLV3-9) compared with other less broad antibodies such as CC12.3 (IGKV3-20), which may affect the overall binding interactions; however, our analysis indicates that there is less hydrogen bonding between the S728-1157 light chain and the RBD compared with CC12.3 (Supplemental Table 7). Although most of the CDR-H3 contact residues critical for VOC cross-reactivity in this interaction are germline-encoded and not introduced by somatic mutations, several somatically mutated residues in framework regions or CDR-H1, CDR-H2, and CDR-L1 are involved in interaction with SARS-CoV-2 RBD. On the one hand, this suggests that memory B cells encoding IGHV3-53/66 class antibodies could acquire a similar degree of cross-reactivity by further affinity maturation. On the other hand, this also indicates the possibility of designing germline-targeted immunogens that target S728-1157-like naive B cells. While it may be challenging to design vaccines that can specifically elicit S728-1157–like antibodies with select CDR-H3s capable of overcoming the VOC mutations, it is encouraging that IGHV-gene restriction is observed in other potent SARS-CoV-2 neutralizing mAbs studies (13, 15, 20–27). Alternatively, this may also be feasible through iterative immunization with optimized RBD immunogens, as has been previously reported for other pathogens (51–55).

Although many mutations have been observed in the RBS-A/class 1 antigenic site (18), with regard to the S728-1157 epitope, 13 of 15 total RBD contact residues and 2 of 3 CDR-H3-bound RBD contact residues are conserved within Omicron and all other VOCs. This suggests that the RBD region where the S728-1157 epitope is found may include residues critical for its dynamic function and viral fitness and would therefore be less tolerant of mutations and antigenic drift than surrounding RBS-A/ class 1 site residues. If this is the case, the tendency for this particular epitope to be lost as viral variants evolve should be reduced, making characterization of S728-1157 and similar antibodies and epitopes important for variant-resistant vaccines or mAb therapeutic development.

In summary, our study identifies bnAbs that may inform immunogen design for next-generation variant-proof coronavirus vaccines or serve as mAb therapeutics that are resistant to SARS-CoV-2 evolution. In particular, in terms of combined potency and breadth, S728-1157 appears to be the best-in-class antibody isolated to date. Given that this antibody binds more readily with 6P-stabilization, it is predicted to be preferentially induced by 6P-stabilized recombinant spike proteins or whole virus, which suggests that hexaproline modification could benefit future vaccine constructs in order to optimally protect against future SARS-CoV-2 variants and other sarbecoviruses.

## Methods

### Monoclonal antibody isolation.

PBMCs were isolated from leukoreduction filters and frozen as described previously (24). B cells were enriched from PBMCs via FACS. Cells were stained with CD19, CD3, and antigen probes conjugated to oligo-fluorophore; cells of interest were identified as CD3^–^CD19^+^Antigen^+^. All mAbs were generated from oligo-tagged, antigen bait-sorted cells identified through single-cell RNA-Seq, as described previously (15, 24). The single B cell data generated in this study have been deposited to Gene Expression Omnibus: GSE171703 and GSM5231088–GSM5231123.

Antigen-specific B cells were selected to generate mAbs based on antigen-probe intensity analyzed by JMP Pro 15. Antibody heavy and light chain genes were synthesized by Integrated DNA Technologies (IDT) and cloned into human IgG1 and human κ or λ light-chain expression vectors by Gibson assembly, as previously described (56). The heavy and light chains of the corresponding mAb were transiently cotransfected into HEK293T cells (ATCC). After transfection for 18 hours, the transfected cells were supplemented with protein-free hybridoma medium supernatant (PFHM-II, Gibco). The supernatant containing secreted mAb was harvested at day 4 and purified using protein A-agarose beads (Thermo Fisher Scientific) as detailed previously (56). Sequences of heavy and light chains of the well-characterized antibodies were derived from Protein Data Bank (PDB), LY-CoV555 (PDB ID: 7KMG), CR3022 (PDB ID: 6W7Y), and REGN10933 (PDB ID: 6XDG) and were synthesized as described above. The CC12.3 mAb (PDB ID: 6XC4) was provided by Meng Yuan at the Scripps Research Institute (San Diego, California, USA).

### Recombinant spike protein expression.

The recombinant D614G SARS-CoV-2 full-length (FL) spike, BA.2-6P, BA.4/5-6P, BQ.1-6P, BQ.1.1-6P, XBB-6P, WT RBD, single RBD mutants (R346S, K417N, K417T, G446V, L452R, S477N, F486A, F486Y, N487Q, Y489F, Q493A, Q493N, N501Y, Y505A, and Y505F), combination RBD mutant (K417N/E484K/L452R/NN501Y), SARS-CoV-1 RBD, and MERS-CoV RBD were generated in-house. Briefly, the recombinant antigens were expressed using Expi293F cells (Thermo Fisher Scientific). The gene of interest was cloned into a mammalian expression vector (in-house modified AbVec) and transfected using the ExpiFectamine 293 kit (Thermo Fisher Scientific) according to the manufacturer’s protocol. The supernatant was harvested at day 4 after transfection and incubated with Ni-nitrilotriacetic acid (Ni-NTA) agarose (Qiagen). The purification was carried out using a gravity flow column and eluted with imidazole-containing buffer as previously described (57, 58). The eluate was buffering-exchanged with PBS using Amicon centrifugal unit (Millipore). The recombinant FL spikes stabilized by 2P mutations of the variants B.1.1.7 Alpha, B.1.351 Beta, P.1 Gamma, B.1.617.2 Delta, BA.1, BA.2, and BA.4 Omicron and were produced in the Sather Laboratory at Seattle Children’s Research Institute. The K417V, N439K, and E484K RBDs and recombinant FL spike WT-2P and 6P were produced in Krammer laboratory at the Icahn School of Medicine at Mount Sinai. The SARS-CoV-2-6P-Mut7 and spike BA.1-6P were designed and produced as described in a previous study (59). The protein sequences and resources for each antigen are listed in Supplemental Table 4[Sec sd].

### ELISA.

Recombinant SARS-CoV-2 spike/RBD proteins were coated onto high protein–binding microtiter plates (Costar) at 2 μg/mL in PBS at 50 μL/well, and kept overnight at 4°C. Plates were washed with PBS containing 0.05% Tween 20 (PBS-T) and blocked with 150 μL of PBS containing 20% FBS for 1 hour at 37°C. Monoclonal antibodies were serially diluted 3-fold starting from 10 μg/mL in PBS and incubated in the wells for 1 hour at 37°C. Plates were then washed and incubated with HRP-conjugated goat anti-human IgG antibody (Jackson ImmunoResearch; 109-035-098), 1:1,000) for 1 hour at 37°C. After washing, 100 μL of Super AquaBlue ELISA substrate (eBioscience) was added per well. Absorbance was measured at 405nm on a microplate spectrophotometer (Bio-Rad). The assays were standardized using control antibody S144-509 (15), with known binding characteristics in every plate, and the plates were developed until the absorbance of the control reached an OD of 3.0. All mAbs were tested in duplicate, and each experiment was performed twice.

### Serum ELISA.

High protein–binding microtiter plates were coated with recombinant SARS-CoV-2 spike antigens at 2 μg/mL in PBS overnight at 4°C. Plates were washed with PBS 0.05% Tween and blocked with 200 μL PBS 0.1% Tween + 3% skim milk powder for 1 hour at room temperature (RT). Plasma samples were heat-inactivated for 1 hour at 56°C before performing the serology experiment. Plasma were serially diluted 2-fold in PBS 0.1% Tween + 1% skim milk powder. Plates were incubated with serum dilutions for 2 hours at RT. The HRP-conjugated goat anti-human Ig secondary antibody diluted at 1:3,000 with PBS 0.1% Tween + 1% skim milk powder was used to detect binding of antibodies. After 1 hour of incubation, plates were developed with 100 μL SigmaFast OPD solution (Sigma-Aldrich) for 10 minutes. Then, 50 μL 3M HCl was used to stop the development reaction. Absorbance was measured at 490 nm on a microplate spectrophotometer (Bio-Rad). End point titers were extrapolated from sigmoidal 4PL (where *x* is log concentration) standard curve for each sample. Limit of detection (LOD) is defined as the mean + 3 SD of the OD signal recorded using plasma from preSARS-CoV-2 individuals. All calculations were performed in GraphPad Prism software (version 9.0).

### Competition ELISA.

To determine the target epitope classification of RBD-reactive mAbs, competition ELISAs were performed using other mAbs with known epitope binding characteristics as competitor mAbs. Competitor mAbs were biotinylated using EZ-Link sulfo-NHS-biotin (Thermo Fisher Scientific) for 2 hours at RT. The excess biotin of biotinylated mAbs was removed with 7k molecular weight-cutoff (MWCO) Zeba spin desalting columns (Thermo Fisher Scientific). Plates were coated with 2 μg/mL RBD antigen overnight at 4°C. Plates were blocked with PBS–20% FBS for 2 hours at RT, and the 2-fold dilution of the mAbs of an undetermined class or serum, were added, starting at 20 μg/mL of mAbs and a 1:10 dilution of serum. After antibody incubation for 2 hours at RT, the biotinylated competitor mAb was added at a concentration twice that of its dissociation constant (K_D_) and incubated for another 2 hours at RT together with the mAb or serum that was previously added. Plates were washed and incubated with 100 μL HRP-conjugated streptavidin (Southern Biotech) at a dilution of 1:1,000 for 1 hour at 37°C. The plates were developed with the Super AquaBlue ELISA substrate (eBioscience). To normalize the assays, the competitor biotinylated mAb was added in a well without any competing mAbs or serum as a control. Data were recorded when the absorbance of the control well reached and OD of 1.0–1.5. The percent competition between mAbs was then calculated by dividing a sample’s observed OD by the OD reached by the positive control, subtracting this value from 1, and multiplying by 100. For serum, ODs were log_10_ transformed and analyzed by nonlinear regression to determine the 50% inhibition concentration (IC_50_) values using GraphPad Prism software (version 9.0). The data were transformed to Log1P and plotted into graph representative of reciprocal serum dilution of the IC_50_ of serum dilution that can achieve 50% competition with the competitor mAb of interest. All mAbs were tested in duplicate, each experiment was performed 2 times independently, and values from 2 independent experiments were averaged.

### Plaque assays.

Plaque assays were performed with SARS-CoV-2 variant viruses on Vero E6/TMPRSS2 cells (Japanese Collection of Research Bioresources (JCRB)) (Supplemental Table 5). Cells were cultured to achieve 90% confluency before being trypsinized and seeded at a density of 3 × 10^4^ cells/well in 96-well plates. On the following day, 10^2^ PFUs of SARS-CoV-2 variant were incubated with 2-fold-diluted mAbs for 1 hour. The antibody-virus mixture was incubated with Vero E6/TMPRSS2 cells for 3 days at 37°C. Plates were fixed with 20% methanol and then stained with crystal violet solution. The complete inhibitory concentrations (IC_99_) were calculated using the log(inhibitor) versus normalized response (variable slope), performed in GraphPad Prism (version 9.0). All mAbs were tested in duplicate, and each experiment was performed twice.

### Focus reduction neutralization test.

Focus reduction neutralization tests (FRNTs) were used to determine neutralization activities as an additional platform aside from the plaque assay. Serial dilutions of serum starting at a final concentration of 1:20 were mixed with 10^3^ focus-forming units of virus per well and incubated for 1 hour at 37 °C. A pooled prepandemic serum sample served as a control. The antibody-virus mixture was inoculated onto Vero E6/TMPRSS2 cells (JCRB) in 96-well plates and incubated for 1 hour at 37 °C. An equal volume of methylcellulose solution was added to each well. The cells were incubated for 16 hours at 37°C and then fixed with formalin. After the formalin was removed, the cells were immunostained with a mouse mAb against SARS-CoV-1/2 nucleoprotein [clone 1C7C7 (Sigma-Aldrich)], followed by a HRP-labeled goat anti-mouse immunoglobulin (Sigma-Aldrich; A8924). The infected cells were stained with TrueBlue Substrate (SeraCare Life Sciences) and then washed with distilled water. After drying, the focus numbers were quantified by using an ImmunoSpot S6 Analyzer, ImmunoCapture software, and BioSpot software (Cellular Technology). The IC_50_ was calculated from the interpolated value from the log(inhibitor) versus normalized response, using variable slope (4 parameters) nonlinear regression performed in GraphPad Prism (version 9.0).

### Negative stain electron microscopy

Spike BA.1 Omicron-6P was complexed with a 0.5-fold M excess of IgG S728-1157 and incubated for 30 minutes at RT. The complex was diluted to 0.03 mg/mL and deposited on a glow-discharged carbon-coated copper mesh grid. 2% uranyl formate (w/v) was used to stain the sample for 90 seconds. The negative stain data set was collected on a Thermo Fisher Tecnai T12 Spirit (120keV, 56,000 × magnification, 2.06 apix) paired with a FEI Eagle 4k × 4k CCD camera. Leginon(60) was used to automate the data collection and raw micrographs were stored in the Appion database (61). Dogpicker (62) picked particles and the data set was processed in RELION 3.0 (62). UCSF Chimera (63) was used for map segmentation and figure making.

#### Cryo-electron microscopy and model building.

SARS-CoV-2-6P-Mut7 was complexed with a 0.5-fold molar excess of IgG S728-1157 relative to trimer (3 binding sites) and incubated for 30 minutes at RT. Grids were prepared using a Thermo Fisher Vitrobot Mark IV set to 4°C and 100% humidity. The complex, at 0.7 mg/mL, was briefly incubated with lauryl maltose neopentyl glycol (final concentration of 0.005 mM; Anatrace), deposited on a glow-discharged Quantifoil 1.2/1.3-400 mesh grid, and blotted for 3 seconds. The grid was loaded into a Thermo Fisher Titan Krios (130,000 × magnification, 300 kEV, 1.045-Å pixel size) paired with a Gatan 4k × 4k K2 Summit direct electron detector. The Leginon software was used for data collection automation and resulting images were stored in the Appion database. Initial data processing was performed with cryoSPARC v3.2 (64), which included CTF correction using GCTF (65), template picking, and 2D and 3D classification and refinement methods leading to an approximately 3.3 Å C1 global reconstruction. The particles from this reconstruction were imported into Relion 3.1 (66), subjected to C3 symmetry expansion followed by focused 3D classifications without alignments using a mask around the antibody Fab and S-protein RBD regions of a single protomer. Classes with well-resolved density in this region were selected and subjected to additional rounds of focused classification. Refinements were performed with limited angular searches and a mask around the trimeric S-protein and a single Fab. The final set of particles reconstructed to approximately 3.7 Å global resolution.

Model building was initiated by rigid body docking of the x-ray structure of the Fab and a published cryo-EM model of the SARS-CoV-2 spike open state (PDB ID: 6VYB) into the cryo-EM map using UCSF Chimera (63). Manual building, mutagenesis, and refinement were performed in Coot 0.9.6 (67), followed by relaxed refinement using Rosetta Relax (68). Model manipulation and validation was also done using Phenix 1.20 (69). Data collection, processing, and model building statistics are summarized in Supplemental Table 8. Figures were generated using UCSF ChimeraX (70).

#### Crystallization and X-ray structure determination.

We used 384 conditions of the JCSG Core Suite (Qiagen) for crystal screening of S728-1157 Fab crystals on the robotic CrystalMation system (Rigaku) at Scripps Research. Crystallization trials were set up by the vapor diffusion method in sitting drops containing 0.1 μL of protein complex and 0.1 μL of reservoir solution. Crystals appeared on day 14, were harvested on day 21, preequilibrated in cryoprotectant containing 15% ethylene glycol, and flash cooled and stored in liquid nitrogen until data collection. Diffraction quality crystals were obtained in solution containing 0.2 M diammonium tartrate, and 20% (w/v) polyethylene glycol (PEG) 3350. Diffraction data were collected at cryogenic temperature (100 K) on Scripps/Stanford beamline 12-1 at the Stanford Synchrotron Radiation Lightsource (SSRL). The X-ray data were processed with HKL2000 (71). The X-ray structures were solved by molecular replacement (MR) using PHASER (72) with MR models for the Fabs from PDB ID: 7KN4 (73). Iterative model building and refinement were carried out in COOT (74) and PHENIX (75), respectively. (76)

#### Animals and challenge viruses.

To determine whether mAbs in the panel could reduce viral load in vivo, female, 6–8 week-old Syrian hamsters (Envigo) were intraperitoneally administered 5 mg/kg of candidate mAb 1 day after intranasal infection with 10^3^ PFU of SARS-CoV-2 viruses (an early SARS-CoV-2 isolate, Delta, or BA.1 Omicron). Control animals were treated with an Ebola-specific mAb (KZ52) of matched isotype. At day 4 after infection, lung tissues and the nasal turbinate were collected to evaluate viral titers by standard plaque assay on Vero E6/TMPRRSS2 cells (JCRB). The animal study was conducted in accordance with the recommendations for care and use of animals by the IACUC at the University of Wisconsin under BSL-3 containment using approved protocols.

#### Biolayer interferometry.

To determine precise binding affinity, the dissociation constant (K_D_) of each mAb was performed by biolayer interferometry (BLI) with an Octet K2 instrument (Forte Bio/Sartorius). The trimeric spike SARS-CoV-2 and its variants were biotinylated (EZ-Link Sulfo-NHS-Biotin, Thermo Fisher Scientific), desalted (Zeba Spike Desalting, Thermo Fisher Scientific), and loaded at a concentration of 500 nM onto streptavidin (SA) biosensor (Forte Bio/Sartorius) for 300 seconds, followed by kinetic buffer (1 × PBS containing 0.02% Tween-20 and 0.1% BSA) for 60 s. The biosensor was then moved to associate with mAbs of interest (142 nM) for 300 seconds, followed by disassociation with the kinetic buffer for 300 seconds. On rate, off rate, and K_D_ were evaluated with a global fit, the average of those values with high R-squared from 2 independent experiments were presented. Analysis was performed by Octet Data Analysis HT software (Forte Bio/Sartorius) with 1:1 fitting model for Fabs and 1:2 interacting model for IgG.

For competitive assay by BLI, streptavidin (SA) biosensor was preequilibrated in 1 × PBS for at least 600 seconds to bind with the biotinylated trimeric spike WT-6P and spike BA.1 Omicron-6P for 300 seconds. The first mAb was associated on the loaded sensor for 300 seconds, followed by the second mAb for another 300 seconds. The final volume for all the solutions was 200 μL/well. All of the assays were performed with kinetic buffer at 30°C. Data were analyzed by Octet Data Analysis HT software (Forte Bio/Sartorius) and plotted using GraphPad Prism.

#### Statistics.

All statistical analyses were performed using GraphPad Prism software (version 9.0). The numbers of biological repeats for experiments and specific tests for statistical significance used are described in the corresponding figure legends. *P* ≤ 0.05 was considered significant. Tukey’s multiple pairwise–comparison was utilized to determine the difference of the proportion of antigen-specific clones in the convalescent cohort. The non-parametric Kruskal-Willis test followed by Dunn’s multiple comparison tests were used to compare somatic hypermutation rates and serum ELISA IC_50_. Wilcoxon matched–pairs signed rank test was used for the comparison of paired serum antibody titers between two antigens from the same individual/timepoint, as well as between timepoints from the same individuals. Viral titers were compared using unpaired 2-tailed *t* tests.

#### Study approvals.

For mAb production, human PBMCs and serum of the convalescent cohort were collected during the first wave of the pandemic in May 2020, before other SARS-CoV-2 variants emerged, which is outlined in Supplemental Table 1. All studies were performed with the approval of the University of Chicago IRB (IRB20-0523). All participants provided prior written informed consent for the use of blood in research applications. This clinical trial was registered at ClinicalTrials.gov under identifier NCT04340050. For serum competition ELISA, plasma from the mRNA-vaccination cohort were collected from participants in the longitudinal observational study under program PARIS. All PARIS participants provided written consent prior to study participation. The study was approved by the Mount Sinai Hospital Institutional Review Board (IRB-20-03374) and further details are outlined in Supplemental Table 2A and Supplemental Table 2B.

## Author contributions

SC and PCW conceptualized the study. SC, PJH, HL, and JLT conducted the experiments, acquired and analyzed data, and wrote the manuscript. JJCM, GO, LL, GDW, MK, TM, MH, NYZ, HLT, SAE, YF, AY, and GS performed experiments and analyzed data. BM, JM, KS, and VS provided biospecimens. JJCM, GDW, BM, and VS edited the manuscript. FK, DNS, ABW, IAW, and YK provided funding and resources. PCW, IAW, ABW, and YK supervised the work, provided critical insights, and wrote the manuscript.

## Supplementary Material

Supplemental data

## Version 1 03/02/2023

In-Press Preview

## Version 2 04/17/2023

Electronic publication

## References

[1] Hou YJ (2020). SARS-CoV-2 D614G variant exhibits efficient replication ex vivo and transmission in vivo. Science.

[2] Garcia-Beltran WF (2021). Multiple SARS-CoV-2 variants escape neutralization by vaccine-induced humoral immunity. Cell.

[3] Wall EC (2021). Neutralising antibody activity against SARS-CoV-2 VOCs B.1.617.2 and B.1.351 by BNT162b2 vaccination. Lancet.

[4] Edara VV (2021). Infection and vaccine-induced neutralizing-antibody responses to the SARS-CoV-2 B.1.617 variants. N Engl J Med.

[5] Zhou D (2021). Evidence of escape of SARS-CoV-2 variant B.1.351 from natural and vaccine-induced sera. Cell.

[6] Weisblum Y (2020). Escape from neutralizing antibodies by SARS-CoV-2 spike protein variants. Elife.

[7] Graham F (2021). Daily briefing: Omicron coronavirus variant puts scientists on alert. Nature.

[8] Karim SSA, Karim QA (2021). Omicron SARS-CoV-2 variant: a new chapter in the COVID-19 pandemic. Lancet.

[9] Carreño JM (2021). Activity of convalescent and vaccine serum against SARS-CoV-2 Omicron. Nature.

[10] Wang Q (2022). Alarming antibody evasion properties of rising SARS-CoV-2 BQ and XBB subvariants. Cell.

[11] VanBlargan LA (2022). An infectious SARS-CoV-2 B.1.1.529 Omicron virus escapes neutralization by therapeutic monoclonal antibodies. Nat Med.

[12] Takashita E (2022). Efficacy of antibodies and antiviral drugs against Covid-19 Omicron Variant. N Engl J Med.

[13] Yuan M (2021). Recognition of the SARS-CoV-2 receptor binding domain by neutralizing antibodies. Biochem Biophys Res Commun.

[14] Barnes CO (2020). SARS-CoV-2 neutralizing antibody structures inform therapeutic strategies. Nature.

[15] Changrob S (2021). Cross-neutralization of emerging SARS-CoV-2 variants of concern by antibodies targeting distinct epitopes on spike. Mbio.

[16] Guthmiller JJ (2021). SARS-CoV-2 infection severity is linked to superior humoral immunity against the spike. Mbio.

[17] Greaney AJ (2021). Mapping mutations to the SARS-CoV-2 RBD that escape binding by different classes of antibodies. Nat Commun.

[18] Liu H, Wilson IA (2022). Protective neutralizing epitopes in SARS-CoV-2. Immunol Rev.

[19] Jette CA (2021). Broad cross-reactivity across sarbecoviruses exhibited by a subset of COVID-19 donor-derived neutralizing antibodies. Cell Rep.

[20] Brouwer PJM (2020). Potent neutralizing antibodies from COVID-19 patients define multiple targets of vulnerability. Science.

[21] Pinto D (2020). Cross-neutralization of SARS-CoV-2 by a human monoclonal SARS-CoV antibody. Nature.

[22] Robbiani DF (2020). Convergent antibody responses to SARS-CoV-2 in convalescent individuals. Nature.

[23] Yuan M (2020). Structural basis of a shared antibody response to SARS-CoV-2. Science.

[24] Dugan HL (2021). Profiling B cell immunodominance after SARS-CoV-2 infection reveals antibody evolution to non-neutralizing viral targets. Immunity.

[25] Rogers TF (2020). Isolation of potent SARS-CoV-2 neutralizing antibodies and protection from disease in a small animal model. Science.

[26] Schmitz AJ (2021). A vaccine-induced public antibody protects against SARS-CoV-2 and emerging variants. Immunity.

[27] Shi R (2020). A human neutralizing antibody targets the receptor-binding site of SARS-CoV-2. Nature.

[28] Cao Y (2020). Potent neutralizing antibodies against SARS-CoV-2 identified by high-throughput single-cell sequencing of convalescent patients’ B cells. Cell.

[29] Barnes CO (2020). Structures of human antibodies bound to SARS-CoV-2 spike reveal common epitopes and recurrent features of antibodies. Cell.

[30] Corbett KS (2020). SARS-CoV-2 mRNA vaccine design enabled by prototype pathogen preparedness. Nature.

[31] Amanat F (2021). Introduction of two prolines and removal of the polybasic cleavage site lead to higher efficacy of a recombinant spike-based SARS-CoV-2 vaccine in the mouse model. mBio.

[32] Sun W (2021). A Newcastle disease virus expressing a stabilized spike protein of SARS-CoV-2 induces protective immune responses. Nat Commun.

[33] Hsieh CL (2020). Structure-based design of prefusion-stabilized SARS-CoV-2 spikes. Science.

[34] Gobeil SM (2022). Structural diversity of the SARS-CoV-2 Omicron spike. Mol Cell.

[35] Yuan M (2020). A highly conserved cryptic epitope in the receptor binding domains of SARS-CoV-2 and SARS-CoV. Science.

[36] Starr TN (2021). Complete map of SARS-CoV-2 RBD mutations that escape the monoclonal antibody LY-CoV555 and its cocktail with LY-CoV016. Cell Rep Med.

[37] Baum A (2020). REGN-COV2 antibodies prevent and treat SARS-CoV-2 infection in rhesus macaques and hamsters. Science.

[38] Wu NC (2020). An alternative binding mode of IGHV3-53 antibodies to the SARS-CoV-2 receptor binding domain. Cell Rep.

[39] Wu Y (2020). A noncompeting pair of human neutralizing antibodies block COVID-19 virus binding to its receptor ACE2. Science.

[40] Yuan M (2021). Structural and functional ramifications of antigenic drift in recent SARS-CoV-2 variants. Science.

[41] Yan Q (2021). Germline IGHV3-53-encoded RBD-targeting neutralizing antibodies are commonly present in the antibody repertoires of COVID-19 patients. Emerg Microbes Infect.

[42] Zhang Q (2021). Potent and protective IGHV3-53/3-66 public antibodies and their shared escape mutant on the spike of SARS-CoV-2. Nat Commun.

[43] Wang Z (2021). mRNA vaccine-elicited antibodies to SARS-CoV-2 and circulating variants. Nature.

[44] Simon V (2022). PARIS and SPARTA: finding the achilles’ heel of SARS-CoV-2. mSphere.

[45] Starr TN (2021). SARS-CoV-2 RBD antibodies that maximize breadth and resistance to escape. Nature.

[46] Walls AC (2020). Structure, function, and antigenicity of the SARS-CoV-2 spike glycoprotein. Cell.

[47] Henderson R (2020). Controlling the SARS-CoV-2 spike glycoprotein conformation. Nat Struct Mol Biol.

[48] Shrestha LB (2021). Broadly-neutralizing antibodies against emerging SARS-CoV-2 Variants. Front Immunol.

[49] Greaney AJ (2021). Antibodies elicited by mRNA-1273 vaccination bind more broadly to the receptor binding domain than do those from SARS-CoV-2 infection. Sci Transl Med.

[50] Reincke SM (2022). SARS-CoV-2 Beta variant infection elicits potent lineage-specific and cross-reactive antibodies. Science.

[51] Wrammert J (2011). Broadly cross-reactive antibodies dominate the human B cell response against 2009 pandemic H1N1 influenza virus infection. J Exp Med.

[52] Guthmiller JJ (2021). First exposure to the pandemic H1N1 virus induced broadly neutralizing antibodies targeting hemagglutinin head epitopes. Sci Transl Med.

[53] Bajic G (2019). Influenza antigen engineering focuses immune responses to a subdominant but broadly protective viral epitope. Cell Host Microbe.

[54] Nachbagauer R (2021). A chimeric hemagglutinin-based universal influenza virus vaccine approach induces broad and long-lasting immunity in a randomized, placebo-controlled phase I trial. Nat Med.

[55] Angeletti D (2019). Outflanking immunodominance to target subdominant broadly neutralizing epitopes. Proc Natl Acad Sci U S A.

[56] Guthmiller JJ (2019). An efficient method to generate monoclonal antibodies from human B Cells. Methods Mol Biol.

[57] Amanat F (2020). A serological assay to detect SARS-CoV-2 seroconversion in humans. Nat Med.

[58] Stadlbauer D (2020). SARS-CoV-2 seroconversion in humans: a detailed protocol for a serological assay, antigen production, and test setup. Curr Protoc Microbiol.

[59] Torres JL (2022). Structural insights of a highly potent pan-neutralizing SARS-CoV-2 human monoclonal antibody. Proc Natl Acad Sci U S A.

[60] Suloway C (2005). Automated molecular microscopy: the new Leginon system. J Struct Biol.

[61] Lander GC (2009). Appion: an integrated, database-driven pipeline to facilitate EM image processing. J Struct Biol.

[62] Voss NR (2009). DoG Picker and TiltPicker: software tools to facilitate particle selection in single particle electron microscopy. J Struct Biol.

[63] Pettersen EF (2004). UCSF Chimera--a visualization system for exploratory research and analysis. J Comput Chem.

[64] Punjani A (2020). Non-uniform refinement: adaptive regularization improves single-particle cryo-EM reconstruction. Nat Methods.

[65] Zhang K (2016). Gctf: Real-time CTF determination and correction. J Struct Biol.

[66] Zivanov J (2018). New tools for automated high-resolution cryo-EM structure determination in RELION-3. Elife.

[67] Casanal A (2020). Current developments in coot for macromolecular model building of electron cryo-microscopy and crystallographic data. Protein Sci.

[68] Frenz B (2019). Automatically fixing errors in glycoprotein structures with rosetta. Structure.

[69] Klaholz BP (2019). Deriving and refining atomic models in crystallography and cryo-EM: the latest Phenix tools to facilitate structure analysis. Acta Crystallogr D Struct Biol.

[70] Pettersen EF (2021). UCSF ChimeraX: Structure visualization for researchers, educators, and developers. Protein Sci.

[71] Otwinowski Z, Minor W (1997). Processing of X-ray diffraction data collected in oscillation mode. Methods Enzymol.

[72] McCoy AJ (2007). Phaser crystallographic software. J Appl Crystallogr.

[73] Qiang M (2022). Neutralizing antibodies to SARS-CoV-2 selected from a human antibody library constructed decades ago. Adv Sci (Weinh).

[74] Emsley P, Cowtan K (2004). Coot: model-building tools for molecular graphics. Acta Crystallogr D Biol Crystallogr.

[75] Adams PD (2010). PHENIX: a comprehensive Python-based system for macromolecular structure solution. Acta Crystallogr D Biol Crystallogr.

[76] Montiel-Garcia D (2022). Epitope-Analyzer: A structure-based webtool to analyze broadly neutralizing epitopes. J Struct Biol.

