# Supplementary material for: Site of vulnerability on SARS-CoV-2 spike induces broadly protective antibody against antigenically distinct Omicron subvariants
Source: J Clin Invest. 2023 Apr 17;133(8):e166844. doi: 10.1172/JCI166844 (PMC10104900; doi:10.1172/JCI166844)
Supplement: Supplemental data [file jci-133-166844-s108.pdf]

Supplemental materials for

Site of vulnerability on SARS-CoV-2 spike induces broadly protective antibody to antigenically distinct Omicron subvariants

Siriruk Changrob[1,15], Peter J. Halfmann[2,15], Hejun Liu[3,15], Jonathan L. Torres[3,15], Joshua J.C. McGrath[1], Gabriel Ozorowski[3], Lei Li[1], G. Dewey Wilbanks[1], Makoto Kuroda[2], Tadashi Maemura[2], Min Huang[1], Nai-Ying Zheng[1], Hannah L. Turner[3], Steven A. Erickson[4], Yanbin Fu[1], Atsuhiro Yasuhara[1], Gagandeep Singh[5,6], Brian Monahan[6,7], Jacob Mauldin[6,7], Komal Srivastava[6,7], Viviana Simon[5,6,7,8,9], Florian Krammer[5,6,7], D. Noah Sather[10], Andrew B. Ward[3], Ian A. Wilson[3,11], Yoshihiro Kawaoka[2,12,13,14], Patrick C. Wilson[1, 16*]

[1] Drukier Institute for Children's Health, Department of Pediatrics, Weill Cornell Medicine, New York, NY 10021, USA.
[2] Influenza Research Institute, Department of Pathobiological Sciences, School of Veterinary Medicine, University of Wisconsin-Madison, Madison, WI 53711, USA.
[3] Department of Integrative Structural and Computational Biology, The Scripps Research Institute, La Jolla, CA 92037, USA.
[4] University of Chicago Department of Medicine, Section of Rheumatology, Chicago, IL 60637, USA.
[5] Department of Pathology, Molecular and Cell Based Medicine, Icahn School of Medicine at Mount Sinai, New York, NY 10029, USA.
[6] Department of Microbiology, Icahn School of Medicine at Mount Sinai, New York, NY 10029, USA.
[7] Center for Vaccine Research and Pandemic Preparedness, Icahn School of Medicine at Mount Sinai, New York, NY, 10029 USA.
[8] The Global Health and Emerging Pathogens Institute, Icahn School of Medicine at Mount Sinai, New York, NY, 10029 USA.
[9] Division of Infectious Diseases, Department of Medicine, Icahn School of Medicine at Mount Sinai, New York, NY 10029, USA.
[10] Center for Global Infectious Disease Research, Seattle Children's Research Institute, Seattle, WA 98101, USA; Department of Pediatrics, University of Washington, Seattle, Washington, USA; Department of Global Health, University of Washington, Seattle, WA 98105, USA.
[11] The Skaggs Institute for Chemical Biology, The Scripps Research Institute; La Jolla, CA 92037, USA.
[12] Division of Virology, Department of Microbiology and Immunology, Institute of Medical Science, University of Tokyo, 108-8639 Tokyo, Japan.
[13] The Research Center for Global Viral Diseases, National Center for Global Health and Medicine Research Institute, Tokyo 162-8655, Japan.
[14] Pandemic Preparedness, Infection and Advanced Research Center (UTOPIA), University of Tokyo, Tokyo 162-8655, Japan.
[15] These authors contributed equally
[16] Lead Contact.
*Correspondence: pcw4001@med.cornell.edu (P.C.W.)
413 E. 69th Street, Suite 1220, New York, NY 10021
Phone: +1 6469629041

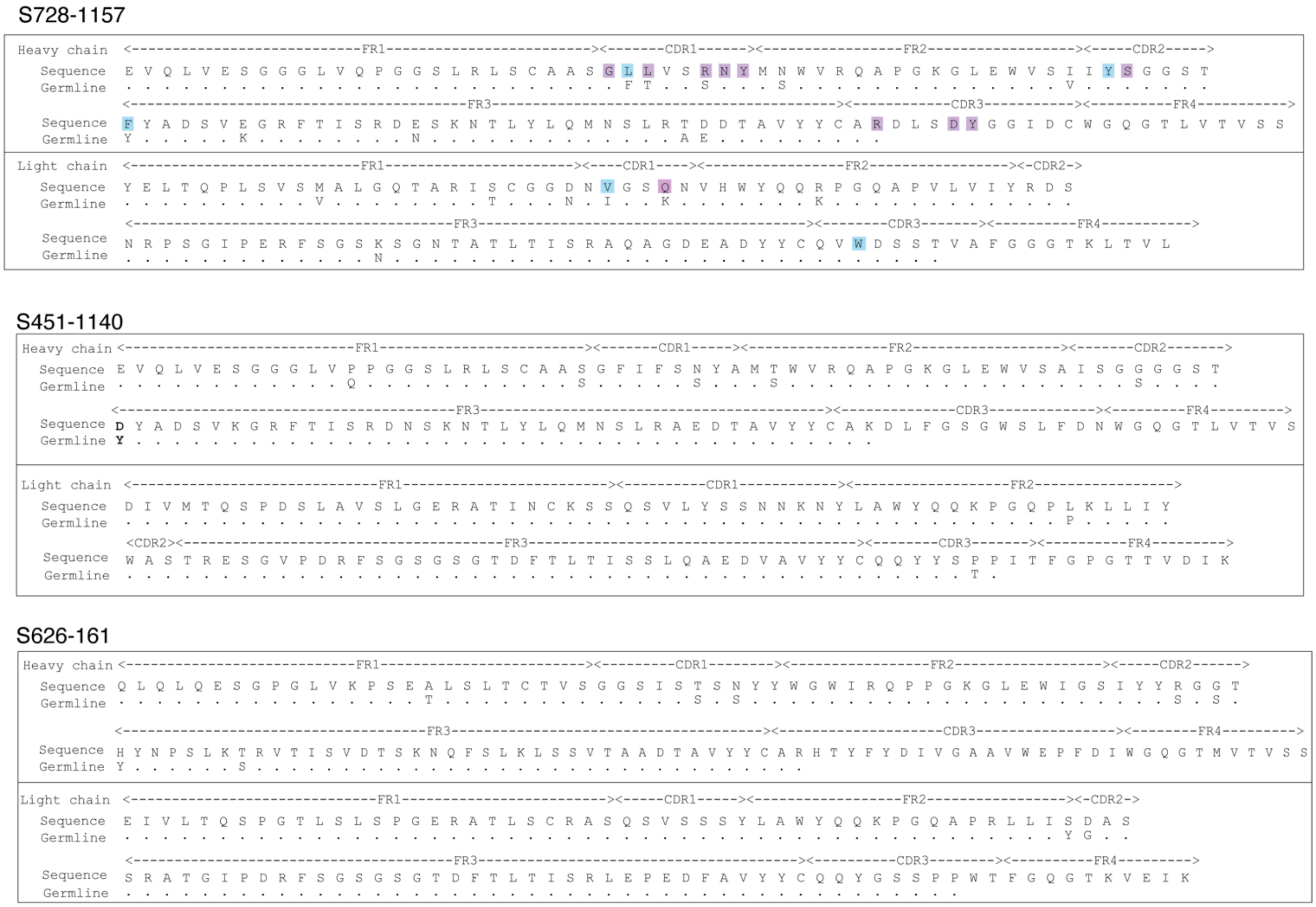

Figure S1. Complementarity-determining region (CDR) sequences of heavy chain and light chain of the three bnAbs. Contacting residues that predicted to make hydrogen bonds between CDR of S728-1157 and SARS-CoV-2 are highlighted with light purple while Van der Waals interactions are highlighted with light blue. Genetic information for each antibody is in **Table S3**.

A

1:2 binding	S728-1157 IgG						S451-1140 IgG						S626-161 IgG					
	Interaction 1			Interaction 2			Interaction 1			Interaction 2			Interaction 1			Interaction 2		
Antigen	k_{on1} ($M^{-1}s^{-1}$)	k_{off1} (s^{-1})	K_{D1} (nM)	k_{on2} ($M^{-1}s^{-1}$)	k_{off2} (s^{-1})	K_{D2} (nM)	k_{on1} ($M^{-1}s^{-1}$)	k_{off1} (s^{-1})	K_{D1} (nM)	k_{on2} ($M^{-1}s^{-1}$)	k_{off2} (s^{-1})	K_{D2} (nM)	k_{on1} ($M^{-1}s^{-1}$)	k_{off1} (s^{-1})	K_{D1} (nM)	k_{on2} ($M^{-1}s^{-1}$)	k_{off2} (s^{-1})	K_{D2} (nM)
WT-6P	2.79E5	1.00E-7	<0.001	2.67E1	5.39E-3	2.02E7	3.75E5	1.00E-7	0.001	1.28E1	1.76E-1	1.38E7	1.13E5	1.90E-4	1.7	3.74E0	5.47E-2	1.46E7
WT-2P	5.43E5	3.94E-4	0.725	4.93E0	1.14E-2	2.31E6	5.63E5	4.92E-4	0.874	1.62E0	1.08E-2	6.65E6	1.49E5	2.82E-4	1.9	6.20E0	3.90E-2	6.30E6
Alpha-2P	1.26E5	3.55E-4	2.8	1.16E1	1.62E-1	1.39E7	1.90E4	6.34E-4	33.3	6.98E0	5.04E-1	7.22E7	5.41E4	5.77E-4	10.7	1.09E1	1.43E-1	1.31E7
Beta-2P	1.76E5	5.88E-4	3.3	4.46E0	7.17E-2	1.61E7	3.03E4	1.38E-3	45.6	3.49E0	2.00E-1	5.71E7	2.14E4	1.15E-4	5.4	3.15E0	2.86E-1	9.09E7
Gamma-2P	8.95E4	4.78E-4	5.3	6.21E0	1.57E-1	2.52E7	2.07E4	6.75E-4	32.6	5.11E0	4.79E-1	9.37E7	3.98E4	8.87E-4	22.3	3.48E0	1.15E-1	3.30E7
Delta-2P	1.75E5	3.47E-4	2.0	1.53E1	9.50E-2	6.20E6	1.91E4	3.12E-4	16.3	4.31E0	4.51E-1	1.09E8	3.44E4	1.33E-3	38.6	8.25E0	3.46E-1	4.19E7
BA.1-6P	4.75E5	3.95E-4	0.8	8.94E0	3.89E-2	4.35E6	4.02E4	2.05E-3	51.0	9.01E1	6.41E-2	7.11E7	4.27E4	6.62E-4	15.5	3.42E0	1.43E-1	4.17E7
BA.1-2P	N/A	N/A	N/A	N/A	N/A	N/A	N/A	N/A	N/A	N/A	N/A	N/A	N/A	N/A	N/A	N/A	N/A	N/A
BA.2-2P	5.55E4	1.46E-3	26.3	9.84E0	2.03E-1	2.06E7	N/A	N/A	N/A	N/A	N/A	N/A	N/A	N/A	N/A	N/A	N/A	N/A
BA.4-2P	N/A	N/A	N/A	N/A	N/A	N/A	N/A	N/A	N/A	N/A	N/A	N/A	N/A	N/A	N/A	N/A	N/A	N/A

B

1:1 binding	S728-1157 Fab			S451-1140 Fab			S626-161 Fab		
Antigen	k_{on} ($M^{-1}s^{-1}$)	k_{off} (s^{-1})	K_D (nM)	k_{on} ($M^{-1}s^{-1}$)	k_{off} (s^{-1})	K_D (nM)	k_{on} ($M^{-1}s^{-1}$)	k_{off} (s^{-1})	K_D (nM)
WT-6P	1.43E5	5.19E-4	5.7	1.66E5	5.40E-7	0.005	1.34E5	1.00E-3	9.6
WT-2P	9.13E4	1.16E-3	19.2	5.04E4	9.19E-5	1.5	8.89E4	4.97E-4	6.9
BA.1-6P	6.40E4	2.61E-3	40.8	3.53E4	6.73E-4	19.1	8.28E4	2.98E-3	36
BA.1-2P	N/A	N/A	N/A	N/A	N/A	N/A	N/A	N/A	N/A

C

	Fold-change of k_a ($M^{-1}s^{-1}$) (normalized with 6P)		Fold-change of k_d (s^{-1}) (normalized with 2P)		Fold-change of K_D (nM) (normalized with 2P)	
	WT (2P vs 6P)		WT (2P vs 6P)		WT (2P vs 6P)	
Antibody	IgG	Fab	IgG	Fab	IgG	Fab
S728-1157	0.51	1.57	3940	2.24	725	3.35
S451-1140	0.67	3.30	4923	170.31	874	295.47
S626-161	0.76	1.51	1.49	0.5	1.12	0.72

Figure S2. Broadly neutralizing RBD-reactive mAbs activity against SARS-CoV-2 and emerging variants. (A) Table illustrating the binding rate and equilibrium constants (k_{on}, k_{off}, and affinity binding K_D) measured by BLI of S728-1157, S451-1140 and S626-161 IgG in response to the panel of SARS-CoV-2 VOCs (either former or current VOCs). (**B)** The binding comparison of Fabs of S728-1157, S451-1140 and S626-161 in response to spike WT and BA.1-6P and 2P constructs. The binding traces of IgG and Fab analyzed by BLI were represented by the 1:2 and 1:1 interaction model, respectively. **C,** The fold-change of binding rate (K_{on}, K_{off}) and binding affinity (K_D) between spike WT-6P and spike WT-2P bound by neutralizing RBD-reactive mAbs, whole IgG form and Fab. Data in **B-C** are representative of two independent experiments, and the data from experiments that have the best fit ($R^2 > 0.90$) are selected for analysis.

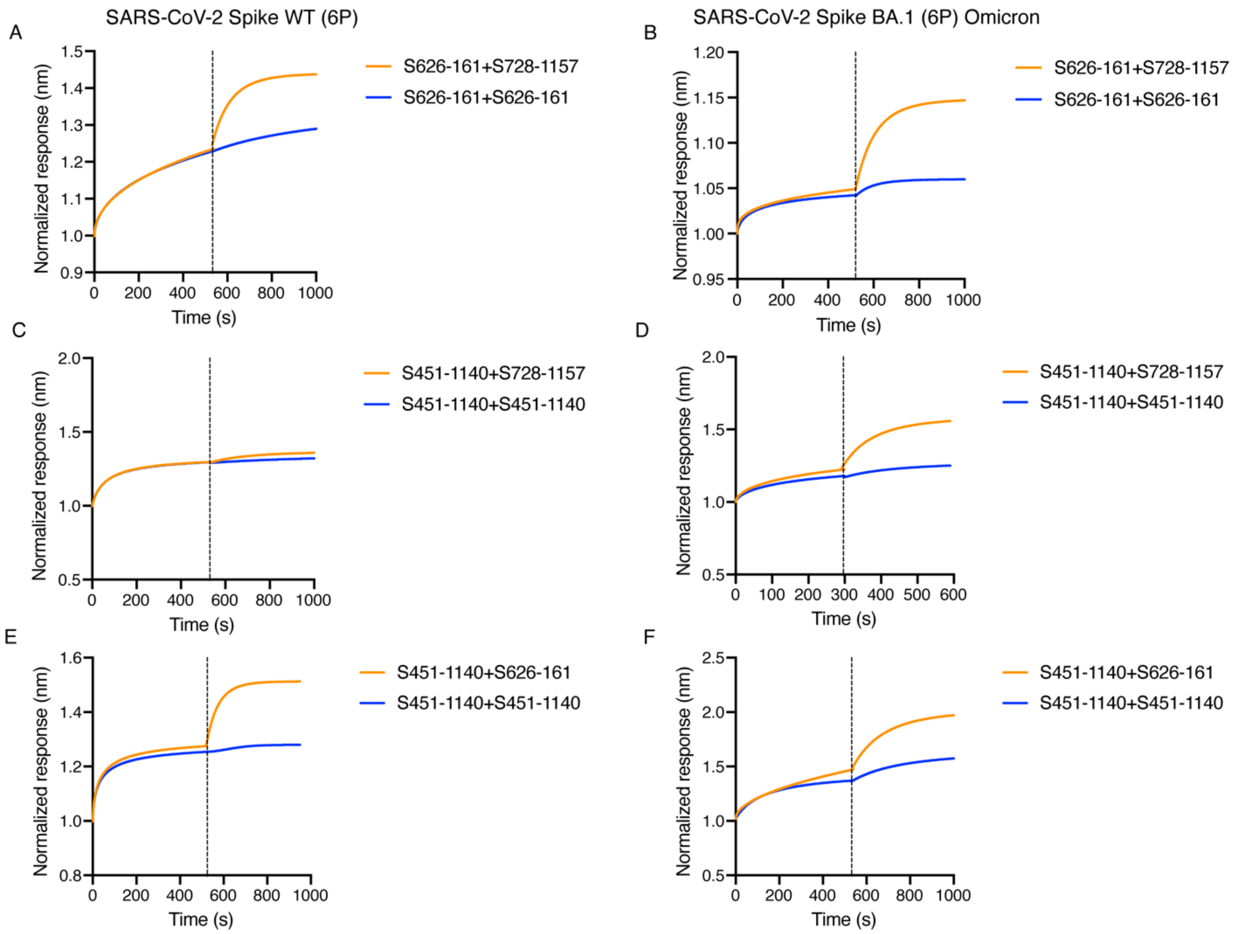

Figure S3. Biolayer interferometry analysis demonstrates binding affinity curves of three broadly neutralizing mAbs competing with each other in response to biotinylated spike wildtype (WT)-6P (left panel) and spike BA.1 Omicron-6P (right panel). (A-B) S626-161 was first bound, followed by S728-1157 mAb as competing mAb. (**C-D)** S451-1140 was first bound and competed with S728-1157 and (**E-F**) S626-161. The response curve was normalized in relation to its starting response value.

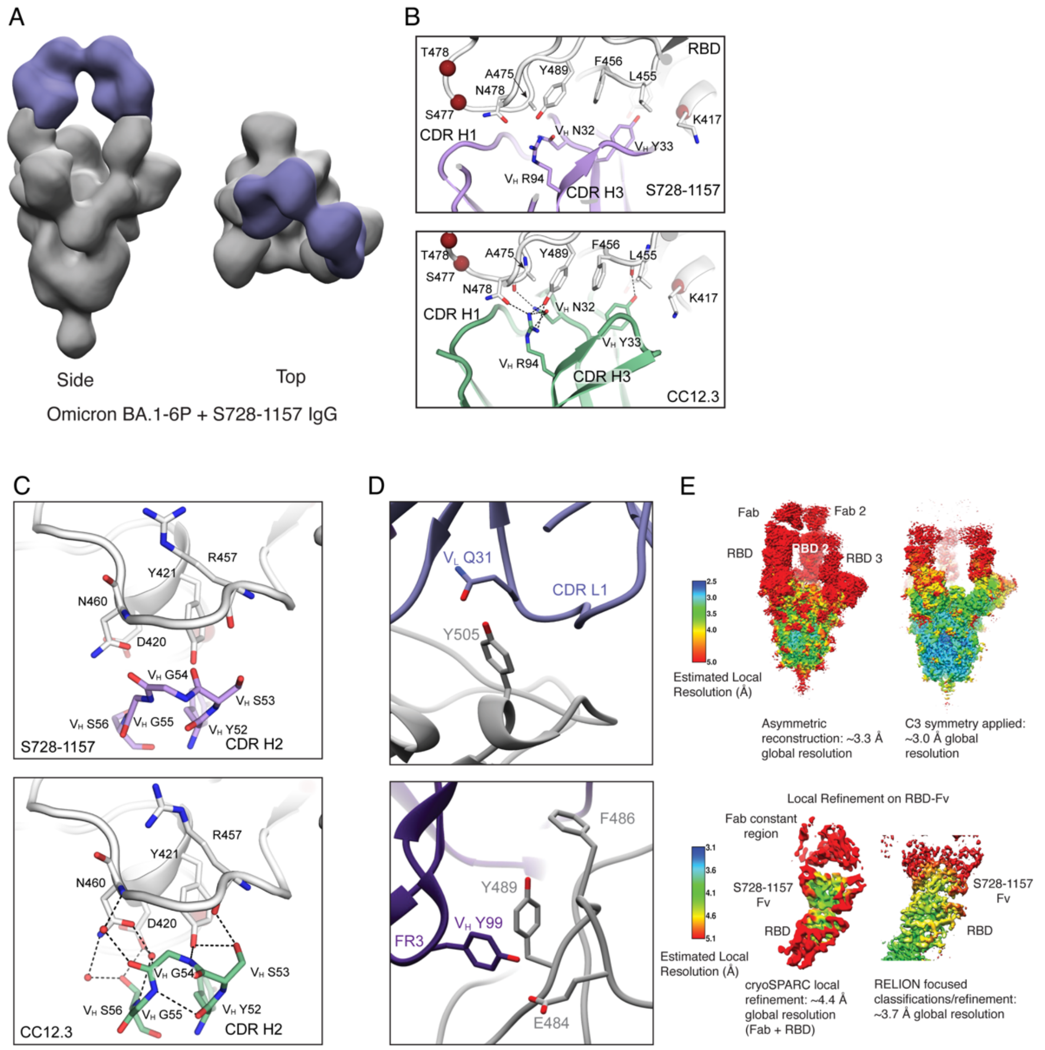

Figure S4. Structural analysis of S728-1157 binding to SARS-CoV-2 spike. (**A)** Three-dimensional (3D) reconstruction of Omicron BA.1-6P in complex with IgG S728-1157 shows binding by negative stain electron microscopy. The binding mode is the same as binding to spike WT-6P-Mut7 shown in **Figure 2B.** (**B)** CDR-H1 of S728-1157 forms similar interactions with SARS-CoV-2 RBD compared to another IGHV3-53 antibody CC12.3 (PDB ID: 6XC4). (**C)** CDR-H2 of S728-1157 forms similar interactions with the RBD compared to CC12.3 (PDB ID: 6XC4). (**D)** For spike WT-6P-Mut7 in complex with S728-1157, residues RBD Y505 and V_L Q31, and E484 and V_H Y99 are predicted to make hydrogen bonds. Hydrophobic residues RBD Y486 and Y489 are also shown. Since S728-1157 binds spike Omicron BA.1-6P in the same way as spike WT-6P-Mut7, it may accommodate the E484A and Y505H mutations in Omicron. (**E)** Local resolution estimates of the cryo-EM map (upper panel) and local refinement on the RBD-Fv after symmetry expansion using RELION (lower panel).

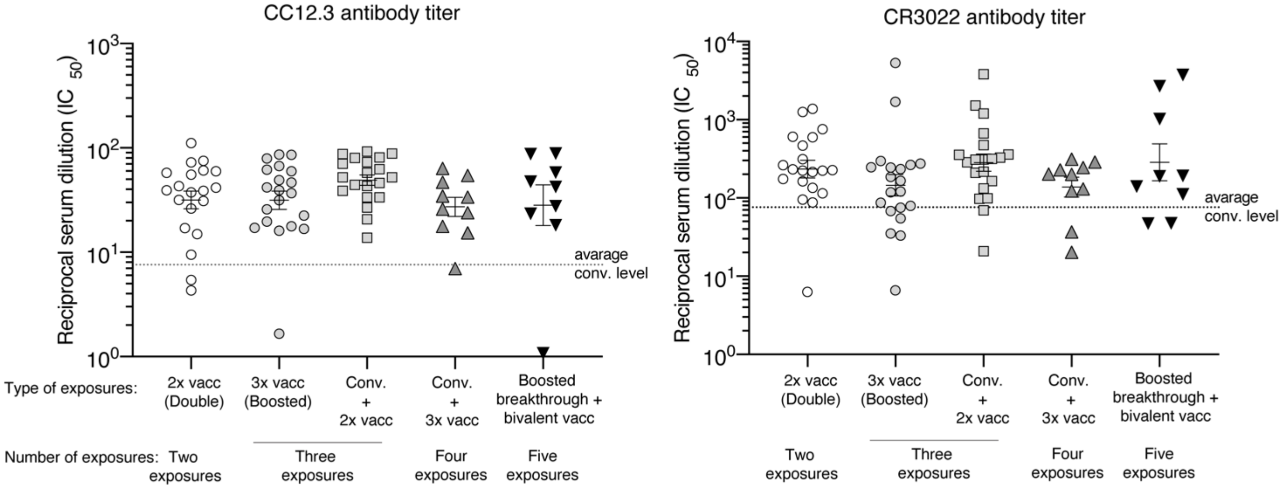

Figure S5. mRNA-vaccinated serum antibody competition with CC12.3 (non-omicron neutralizing class 1 RBD-reactive mAb) and CR3022 (non-neutralizing RBD-reactive mAb). Comparison of IC_{50} of antibody competing for binding to the CC12.3 and CR3022 epitope in plasma from study participants who received mRNA-based vaccines with variety type of exposure history **(A, B)**. 2x vacc, double vaccination (WA-1), (n=20 participants); 3x vacc., boosted or triple vaccination (WA-1) (n=20 participants); conv.+2x vacc., convalescent plus double vaccination (WA-1) (n=20 participants); conv.+3x vacc., convalescent plus boosted/triple vaccination (WA-1) (n=10 participants); boosted breakthrough +bivalent vacc., post-boost infection followed by bivalent vaccination (ancestral, Wuhan-1/BA.5) (n=9 participants). The statistical analysis was determined using Kruskal-Wallis with Dunn's multiple comparison test. No significant differences were left un-indicated in graph. Dashed line indicates average of antibody titer that was found in convalescent individual related to **Figure 5**.

Table S1. COVID-19 convalescent subjects. Related to Figure 1 and Figure 5. The mAbs from high responder subjects, S451, S626, S728 were characterized in this study. Responder group and severity were categorized in a previous study[13]. Serum antibody from each responder group were tested for competition ELISA with broad neutralizing mAbs, other therapeutic mAbs and non-neutralizing mAb.

Subject ID	Age bracket	Sex	SARS-CoV-2 PCR Test	Duration of symptoms (days)	Symptom start to donation (days)	Responder Category[26]	Severity Category[26]
3	20-29	M	3/20	4	33	Low	Moderate
11	60-69	M	3/20	16	49	High	Severe (hospitalized)
17	40-49	M	3/20	17	55	High	Severe
19	50-59	F	3/20	14	44	Low	Moderate
20	30-39	M	3/20	19	48	High	Critical (hospitalized)
22	30-39	F	3/20	3	31	Mid	Moderate
24	30-39	M	3/20	12	41	High	Severe
42	30-39	M	3/20	11	39	Mid	Moderate
63	40-49	M	3/20	2	33	Low	Moderate
80	30-39	M	3/20	12	40	Mid	Moderate
89	60-69	M	3/20	13	43	High	Mild
108	50-59	M	3/20	11	39	High	Moderate
109	30-39	M	3/20	9	41	Low	Moderate
112	40-49	M	3/20	9	40	Low	Moderate
116	60-69	F	3/20	18	49	Low	Moderate
130	50-59	M	3/20	7	35	Mid	Mild
135	20-29	F	3/20	7	36	Low	Moderate
141	60-69	M	3/20	19	48	High	Moderate
144	50-59	M	3/20	23	54	Low	Moderate
156	50-59	F	3/20	11	41	High	Moderate
166	40-49	F	3/20	17	55	Low	Moderate
176	20-29	M	3/20	6	35	Low	Moderate
210	40-49	M	4/20	7	41	Low	Moderate

218	50-59	F	3/20	19	48	Mid	Severe
229	50-59	M	3/20	2	42	Low	Mild
251	50-59	M	3/20	22	51	Low	Severe
266	20-29	F	3/20	4	32	Low	Mild
270	50-59	M	3/20	9	39	Mid	Moderate
272	40-49	M	3/20	14	43	Mid	Moderate
277	60-69	M	3/20	13	45	High	Moderate
278	50-59	F	3/20	12	47	Mid	Moderate
293	70-79	M	3/20	17	63	High	Severe (hospitalized)
305	40-49	F	4/20	4	47	Low	Moderate
319	70-79	M	3/20	4	36	High	Mild
332	30-39	M	3/20	6	35	Mid	Moderate
346	30-39	M	3/20	11	39	Mid	Moderate
355	40-49	F	3/20	14	44	Low	Moderate
373	40-49	M	3/20	7	39	High	Moderate
377	40-49	M	3/20	9	41	High	Moderate
385	30-39	M	3/20	7	47	Mid	Moderate
407	30-39	M	4/20	11	43	Mid	Moderate
433	30-39	M	3/20	6	35	Low	Moderate
447	40-49	M	4/20	21	61	High	Severe
**451**	**40-49**	**M**	**4/20**	**11**	**49**	**High**	**Severe (hospitalized)**
537	30-39	M	3/20	14	59	Mid	Moderate
564	20-29	F	3/20	32	60	Low	Severe
573	20-29	M	3/20	17	56	High	Severe (hospitalized)
**626**	**40-49**	**M**	**3/20**	**19**	**56**	**High**	**Moderate**
**728**	**60-69**	**F**	**3/20**	**53**	**130**	**High**	**Severe**

Table S2A. Demographic, COVID-19 and vaccination data for the PARIS participants Related to **Figure 6** and **Figure S6**. A total of 79 plasma samples were used. Of note, for 31 PARIS participants, samples from two different time points were included (double vaccination and boosted). The plasma samples analyzed were collected, on average 2-4 weeks after each dose of mRNA vaccine.

	Vaccine Immunity		Hybrid Immunity		
	2x Vac	3x Vac	Infection + 2x Vac	Infection + 3x Vax	Bivalent Vac + infection
N total	20	20	20	10	9
Sex at birth					
Male	4 (20%)	5 (25%)	8 (40%)	3 (30%)	2 (22%)
Female	16 (80%)	15 (75%)	12 (60%)	7 (70%)	7 (78.%)
Age					
20-29	4 (20%)	4 (20%)	4 (20%)	2 (20%)	0 (0%)
30-39	5 (25%)	4 (20%)	6 (30%)	3 (30%)	6 (67%)
40-49	6 (30%)	5 (25%)	7 (35%)	1 (10%)	1 (11%)
50-59	4 (20%)	6 (30%)	2 (10%)	2 (20%)	0 (0%)
>60	1 (5%)	1 (5%)	1 (5%)	2 (20%)	2 (22%)
Timing of Infection					
Post-boost infection	n/a	n/a	n/a	n/a	7 (78%)
Pre-vaccine infection	n/a	n/a	20 (100%)	10 (100%)	2 (22%)
No infection	20 (100%)	20 (100%)	n/a	n/a	n/a
Vaccine Type					
Moderna	10 (50%)	10 (50%)	10 (50%)	0 (0%)	0 (0%)
Pfizer	10 (50%)	10 (50%)	10 (50%)	10 (100%)	9 (100%)

Table S2B. Overview of the PARIS participants' samples included in this study.

The ID column provides the participant code. Of note, sequential plasma samples from the same participant are identified by the same ID code by a dash and the number 1, 2 or 3 (e.g., PVI-PW-27-1 and PV1-PW-27-2)

ID	Age	Sex at Birth	Race	Ethnicity	Group #	Number of Doses	Vaccine Type	Timing of Infection
PVI-PW-27-1	45-49	Female	Other	Not Hispanic or Latino	1	2	Pfizer	Negative
PVI-PW-47-1	25-29	Female	Asian	Not Hispanic or Latino	1	2	Pfizer	Negative
PVI-PW-15-1	55-59	Female	Caucasian	Not Hispanic or Latino	1	2	Pfizer	Negative
PVI-PW-33-1	50-54	Female	Caucasian	Not Hispanic or Latino	1	2	Pfizer	Negative
PVI-PW-18-1	25-29	Female	Caucasian	Not Hispanic or Latino	1	2	Pfizer	Negative
PVI-PW-29-1	70-74	Male	Asian	Not Hispanic or Latino	1	2	Pfizer	Negative
PVI-PW-31-1	30-34	Female	Asian Indian	Not Hispanic or Latino	1	2	Pfizer	Negative
PVI-PW-52-1	30-34	Female	Caucasian	Not Hispanic or Latino	1	2	Pfizer	Negative
PVI-PW-9-1	40-44	Male	Caucasian	Not Hispanic or Latino	1	2	Pfizer	Negative
PVI-PW-58-1	45-49	Female	Not Reported	Hispanic or Latino	1	2	Pfizer	Negative
PVI-PW-53	30-34	Female	Caucasian	Not Hispanic or Latino	2	2	Moderna	Negative
PVI-PW-60-1	25-29	Female	Caucasian	Not Hispanic or Latino	2	2	Moderna	Negative
PVI-PW-28-1	40-44	Male	Caucasian	Not Hispanic or Latino	2	2	Moderna	Negative
PVI-PW-34-1	40-44	Female	Caucasian	Not Hispanic or Latino	2	2	Moderna	Negative
PVI-PW-17-1	40-44	Female	Caucasian	Not Hispanic or Latino	2	2	Moderna	Negative
PVI-PW-6-1	35-39	Male	Caucasian	Not Hispanic or Latino	2	2	Moderna	Negative
PVI-PW-43	50-54	Female	Caucasian	Not Hispanic or Latino	2	2	Moderna	Negative
PVI-PW-13-1	55-59	Female	Asian	Not Hispanic or Latino	2	2	Moderna	Negative
PVI-PW-36-1	30-34	Female	Caucasian	Not Hispanic or Latino	2	2	Moderna	Negative
PVI-PW-45-1	20-24	Female	Caucasian	Not Hispanic or Latino	2	2	Moderna	Negative
PVI-PW-27-2	45-49	Female	Other	Not Hispanic or Latino	3	3	Pfizer	Negative
PVI-PW-47-2	25-29	Female	Asian	Not Hispanic or Latino	3	3	Pfizer	Negative
PVI-PW-15-2	55-59	Female	Caucasian	Not Hispanic or Latino	3	3	Pfizer	Negative
PVI-PW-33-2	50-54	Female	Caucasian	Not Hispanic or Latino	3	3	Pfizer	Negative
PVI-PW-18-2	25-29	Female	Caucasian	Not Hispanic or Latino	3	3	Pfizer	Negative
PVI-PW-29-2	70-74	Male	Asian	Not Hispanic or Latino	3	3	Pfizer	Negative
PVI-PW-31-2	30-34	Female	Asian Indian	Not Hispanic or Latino	3	3	Pfizer	Negative
PVI-PW-52-2	30-34	Female	Caucasian	Not Hispanic or Latino	3	3	Pfizer	Negative
PVI-PW-9-2	40-44	Male	Caucasian	Not Hispanic or Latino	3	3	Pfizer	Negative
PVI-PW-58-2	45-49	Female	Not Reported	Hispanic or Latino	3	3	Pfizer	Negative
PVI-PW-60-2	25-29	Female	Caucasian	Not Hispanic or Latino	4	3	Moderna	Negative
PVI-PW-28-2	40-44	Male	Caucasian	Not Hispanic or Latino	4	3	Moderna	Negative
PVI-PW-34-2	40-44	Female	Caucasian	Not Hispanic or Latino	4	3	Moderna	Negative
PVI-PW-17-2	40-44	Female	Caucasian	Not Hispanic or Latino	4	3	Moderna	Negative
PVI-PW-6-2	35-39	Male	Caucasian	Not Hispanic or Latino	4	3	Moderna	Negative
PVI-PW-13-2	55-59	Female	Asian	Not Hispanic or Latino	4	3	Moderna	Negative
PVI-PW-36-2	30-34	Female	Caucasian	Not Hispanic or Latino	4	3	Moderna	Negative
PVI-PW-45-2	20-24	Female	Caucasian	Not Hispanic or Latino	4	3	Moderna	Negative
PVI-PW-4	55-59	Male	Caucasian	Hispanic or Latino	4	3	Moderna	Negative
PVI-PW-5	55-59	Female	Caucasian	Hispanic or Latino	4	3	Moderna	Negative
PVI-PW-2	30-34	Female	Asian	Not Hispanic or Latino	6	4	Pfizer	Post-Boost Infection
PVI-PW-22	45-49	Male	Caucasian	Not Hispanic or Latino	6	4	Pfizer	Post-Boost Infection
PVI-PW-38	35-39	Female	Caucasian	Not Hispanic or Latino	6	4	Pfizer	Post-Boost Infection
PVI-PW-52	30-34	Female	Caucasian	Not Hispanic or Latino	6	4	Pfizer	Post-Boost Infection
PVI-PW-59	60-64	Female	Other	Hispanic or Latino	6	4	Pfizer	Post-Boost Infection
PVI-PW-63	30-34	Female	Caucasian	Not Hispanic or Latino	6	4	Pfizer	Post-Boost Infection
PVI-PW-64	35-39	Female	More than one race	Not Hispanic or Latino	6	4	Pfizer	Post-Boost Infection
PVI-PW-10	40-44	Female	Caucasian	Not Hispanic or Latino	6	4	Moderna	Post-Boost Infection
PVI-PW-60-3	25-29	Female	Caucasian	Not Hispanic or Latino	6	4	Moderna	Post-Boost Infection
PVI-PW-16	45-49	Female	Asian	Not Hispanic or Latino	5A	2	Pfizer	Pre-Vaccine Infection
PVI-PW-20	25-29	Female	Caucasian	Not Hispanic or Latino	5A	2	Pfizer	Pre-Vaccine Infection
PVI-PW-7	30-34	Female	Asian	Not Hispanic or Latino	5A	2	Pfizer	Pre-Vaccine Infection
PVI-PW-1	35-39	Male	Caucasian	Not Hispanic or Latino	5A	2	Pfizer	Pre-Vaccine Infection
PVI-PW-11	40-44	Female	Caucasian	Not Hispanic or Latino	5A	2	Pfizer	Pre-Vaccine Infection
PVI-PW-3	30-34	Female	Caucasian	Not Hispanic or Latino	5A	2	Pfizer	Pre-Vaccine Infection
PVI-PW-12	45-49	Male	Caucasian	Not Hispanic or Latino	5A	2	Pfizer	Pre-Vaccine Infection
PVI-PW-19	30-34	Female	Caucasian	Not Hispanic or Latino	5A	2	Pfizer	Pre-Vaccine Infection
PVI-PW-14	40-44	Male	Latino	Hispanic or Latino	5A	2	Pfizer	Pre-Vaccine Infection
PVI-PW-23	30-34	Female	Caucasian	Not Hispanic or Latino	5A	2	Pfizer	Pre-Vaccine Infection
PVI-PW-8	20-24	Female	Caucasian	Not Hispanic or Latino	5B	2	Moderna	Pre-Vaccine Infection
PVI-PW-24	55-59	Male	Asian	Not Hispanic or Latino	5B	2	Moderna	Pre-Vaccine Infection
PVI-PW-32	30-34	Male	Caucasian	Not Hispanic or Latino	5B	2	Moderna	Pre-Vaccine Infection
PVI-PW-51	20-24	Male	Caucasian	Not Hispanic or Latino	5B	2	Moderna	Pre-Vaccine Infection
PVI-PW-44	25-29	Female	Caucasian	Not Hispanic or Latino	5B	2	Moderna	Pre-Vaccine Infection
PVI-PW-49	60-64	Female	African American	Not Hispanic or Latino	5B	2	Moderna	Pre-Vaccine Infection
PVI-PW-56	40-44	Female	Caucasian	Not Hispanic or Latino	5B	2	Moderna	Pre-Vaccine Infection
PVI-PW-30	45-49	Female	Caucasian	Not Hispanic or Latino	5B	2	Moderna	Pre-Vaccine Infection
PVI-PW-50	55-59	Male	Caucasian	Not Hispanic or Latino	5B	2	Moderna	Pre-Vaccine Infection
PVI-PW-42	45-49	Male	Not Reported	Unknown or Not Reported	5B	2	Moderna	Pre-Vaccine Infection
PVI-PW-37	35-39	Male	Caucasian	Not Hispanic or Latino	5C	3	Pfizer	Pre-Vaccine Infection
PVI-PW-39	30-34	Female	Caucasian	Not Hispanic or Latino	5C	3	Pfizer	Pre-Vaccine Infection
PVI-PW-48	45-49	Female	Not Reported	Unknown or Not Reported	5C	3	Pfizer	Pre-Vaccine Infection
PVI-PW-54	65-69	Male	Caucasian	Not Hispanic or Latino	5C	3	Pfizer	Pre-Vaccine Infection
PVI-PW-40	65-69	Male	Caucasian	Not Hispanic or Latino	5C	3	Pfizer	Pre-Vaccine Infection
PVI-PW-25	25-29	Female	African American	Not Hispanic or Latino	5C	3	Pfizer	Pre-Vaccine Infection
PVI-PW-46	55-59	Female	Caucasian	Not Hispanic or Latino	5C	3	Pfizer	Pre-Vaccine Infection
PVI-PW-21	50-54	Female	Caucasian	Hispanic or Latino	5C	3	Pfizer	Pre-Vaccine Infection
PVI-PW-35	25-29	Female	Caucasian	Not Hispanic or Latino	5C	3	Pfizer	Pre-Vaccine Infection
PVI-PW-57	30-34	Female	Caucasian	Not Hispanic or Latino	5C	3	Pfizer	Pre-Vaccine Infection

Table S3. Characteristics of SARS-CoV-2 RBD-reactive mAbs. Related to Figure 1. Cross-neutralizing mAbs against D614G and B.1.351 Beta, B.1.,617.2 Delta, B.1.617.1 Kappa, B.1.621 Mu, BA.1 Omicron are bolded.

mAb ID	Epitope specificity	VH gene	VL gene	# VH SHM	#VL SHM	CDR-H3 length	CDR-L3 length
S451-5	RBD Class 2	IGHV2-70*01	IGLV1-44*01	4	1	12	11
S451-506	RBD Class 3	IGHV3-53*02	IGKV1-9*01	9	4	12	10
**S451-1140**	**RBD Unclassified**	**IGHV3-23*04**	**IGKV4-1*01**	**8**	**7**	**12**	**9**
S451-1190	RBD Class 3	IGHV2-5*02	IGLV2-14*01	8	8	9	11
S626-84	RBD Class 2	IGHV1-2*02	IGLV2-23*02	7	9	16	10
**S626-161**	**RBD Unclassified**	**IGHV4-39*01**	**IGKV3-20*01**	**8**	**2**	**18**	**10**
S626-664	RBD Unclassified	IGHV4-39*01	IGLV1-51*02	8	5	19	10
S728-209	RBD Unclassified	IGHV2-5*04	IGKV1-12*01	14	12	12	9
S728-369	RBD Unclassified	IGHV4-31*03	IGKV1-5*03	18	13	23	8
S728-430	RBD Class 2	IGHV3-53*01	IGKV1-33*01	1	1	12	10
S728-537	RBD Class 2	IGHV1-2*02	IGKV1-12*01	15	9	17	9
**S728-1157**	**RBD Class 1**	**IGHV3-66*02**	**IGLV3-9*01**	**20**	**9**	**10**	**9**
S728-1261	RBD Unclassified	IGHV4-4*02	IGKV3-20*01	8	12	13	10
S728-1690	RBD Class 2	IGHV1-69*04	IGKV3-20*01	19	8	15	9

Table S4. Antigen information and resource. Proline substitutions are indicated in italics. **Related to Figure 1, Figure 2 and Figure S2.**

Antigen	S1 NTD	RBD	S1 CTD	S2	Source
Spike FL, trimer					
Wildtype(WT)-2P	-	-	-	*K986P, V987P*	Krammer lab
Wildtype(WT)-6P	-	-	-	*F817P, A829P, A899P, A942P, K986P, V987P*	Krammer lab
B.1.1.7 Alpha-2P	del69-70, del144	N501Y	A570D, D614G, P681H	T716I, S982A, *K986P, V987P,* D1118H	Sather lab
B.1.351 Beta-2P	L18F, D80A, D215G, del241-243, R246I	K417N, E484K, N501Y	D614G	A701V, *K986P, V987P*	Sather lab
P.1 Gamma-2P	L18F, T20N, P26S, D138Y, R190S	K417T, E484K, N501Y	D614G, H655Y	*K986P, V987P,* T1027I, V1176F	Sather lab
B.1.617.2 Delta-2P	T19R, G142D, del156-157, R158G	L452R, T478K,	D614G, P681R	D950N, *K986P, V987P*	Sather lab
BA.1 Omicron-2P	A67V, H69del, V70del, T95I, G142D, V143del, Y144del, Y145del, N211del, L212I, insert214EPE	G339D, S371L, S373P, S375F, K417N, N440K, G446S, S477N, T478K, E484A, Q493R, G496S, Q498R, N501Y, Y505H	T547K, D614G, H655Y, N679K, P681H	N764K, D796Y, N856K, Q954H, N969K, L981F, *K986P, V987P*	Sather lab
BA.1 Omicron-6P	A67V, H69del, V70del, T95I, G142D, V143del, Y144del, Y145del, N211del, insert214EPE	G339D, S371L, S373P, S375F, K417N, N440K, G446S, S477N, T478K, E484A,	T547K, D614G, H655Y, N679K, P681H	*V705C,* N764K, D796Y, *F817P, A829P,* N856K, *T883C, A899P, A942P,* Q954H,	Ward lab

		Q493R,<br>G496S,<br>Q498R,<br>N501Y,<br>Y505H		N969K,<br>L981F,<br>*K986P,*<br>*V987P*	
BA.2 Omicron-2P	T19I,<br>L24del,<br>P25del,<br>P26del,<br>A27S,<br>G142D,<br>V213G,	G339D,<br>S371F,<br>S373P,<br>S375F,<br>T376A,<br>D405N,<br>R408S,<br>K417N,<br>N440K,<br>S477N,<br>T478K,<br>E484A,<br>Q493R,<br>Q498R,<br>N501Y,<br>Y505H	D614G,<br>H655Y,<br>N679K,<br>P681H,	N764K,<br>D796Y,<br>Q954H,<br>N969K,<br>*K986P,*<br>*V987P*	Sather lab
BA.2 Omicron-6P	T19I,<br>L24del,<br>P25del,<br>P26del,<br>A27S,<br>G142D,<br>V213G,	G339D,<br>S371F,<br>S373P,<br>S375F,<br>T376A,<br>D405N,<br>R408S,<br>K417N,<br>N440K,<br>S477N,<br>T478K,<br>E484A,<br>Q493R,<br>Q498R,<br>N501Y,<br>Y505H	D614G,<br>H655Y,<br>N679K,<br>P681H,	N764K,<br>D796Y,<br>*F817P,*<br>*A829P,*<br>*A899P,*<br>*A942P*<br>Q954H,<br>N969K,<br>*K986P,*<br>*V987P*	In-house
BA.4/BA.5 Omicron-2P	T19I,<br>L24del,<br>P25del,<br>P26del,<br>A27S,<br>H69del,<br>V70del,<br>G142D,<br>V213G,	G339D,<br>S371F,<br>S373P,<br>S375F,<br>T376A,<br>D405N,<br>R408S,<br>K417N,<br>N440K,<br>L452R,<br>S477N,<br>T478K,<br>E484A,<br>F486V,<br>Q498R,<br>N501Y,<br>Y505H,	D614G,<br>H655Y,<br>N679K,<br>P681H,	N764K,<br>D796Y,<br>Q954H,<br>N969K,<br>*K986P,*<br>*V987P*	Sather lab

BA.4/BA.5 Omicron-6P	T19I,<br>L24del,<br>P25del,<br>P26del,<br>A27S,<br>H69del,<br>V70del,<br>G142D,<br>V213G,	G339D,<br>S371F,<br>S373P,<br>S375F,<br>T376A,<br>D405N,<br>R408S,<br>K417N,<br>N440K,<br>L452R,<br>S477N,<br>T478K,<br>E484A,<br>F486V,<br>Q498R,<br>N501Y,<br>Y505H,	D614G,<br>H655Y,<br>N679K,<br>P681H,	N764K,<br>D796Y,<br>*F817P,*<br>*A829P,*<br>*A899P,*<br>*A942P,*<br>Q954H,<br>N969K,<br>*K986P,*<br>*V987P*	In-house
BQ.1 Omicron-6P<br>(BA.5 + K444T +<br>N460K)	T19I,<br>L24del,<br>P25del,<br>P26del,<br>A27S,<br>H69del,<br>V70del,<br>G142D,<br>V213G,	G339D,<br>S371F,<br>S373P,<br>S375F,<br>T376A,<br>D405N,<br>R408S,<br>K417N,<br>N440K,<br>K444T,<br>L452R,<br>N460K,<br>S477N,<br>T478K,<br>E484A,<br>F486V,<br>Q498R,<br>N501Y,<br>Y505H,	D614G,<br>H655Y,<br>N679K,<br>P681H,	N764K,<br>D796Y,<br>*F817P,*<br>*A829P,*<br>*A899P,*<br>*A942P,*<br>Q954H,<br>N969K<br>*K986P,*<br>*V987P*	In-house
BQ.1.1 Omicron-6P<br>(BA.5 + R346T + K444T<br>+ N460K)	T19I,<br>L24del,<br>P25del,<br>P26del,<br>A27S,<br>H69del,<br>V70del,<br>G142D,<br>V213G,<br>,	G339D,<br>R346T,<br>S371F,<br>S373P,<br>S375F,<br>T376A,<br>D405N,<br>R408S,<br>K417N,<br>N440K,<br>K444T,<br>L452R,<br>N460K,<br>S477N,<br>T478K,<br>E484A,<br>F486V,<br>Q498R,<br>N501Y,<br>Y505H,	D614G,<br>H655Y,<br>N679K,<br>P681H,	N764K,<br>D796Y,<br>*F817P,*<br>*A829P,*<br>*A899P,*<br>*A942P,*<br>Q954H,<br>N969K<br>*K986P,*<br>*V987P*	In-house

XBB Omicron-6P	T19I, L24del, P25del, P26del, A27S, V83A, G142D, Y144del, H146Q, Q183E, V213G,	G339H, R346T, L368I, S371F, S373P, S375F, T376A, D405N, R408S, K417N, N440K, V445P, G446S, N460K, S477N, T478K, E484A, F486S, F490S, Q498R, N501Y, Y505H,	D614G, H655Y, N679K, P681H,	N764K, D796Y, *F817P, A829P, A899P, A942P,* Q954H, N969K, *K986P, V987P*	In-house
RBD					
WT	-	-	-	-	In-house
R346S	-	R346S	-	-	In-house
K417N	-	K417N	-	-	In-house
K417V	-	K417V	-	-	Krammer lab
K417T	-	K417T	-	-	In-house
G446V	-	G446V	-	-	In-house
N439K	-	N439K	-	-	Krammer lab
L452R	-	L452R	-	-	In house
S477N	-	S477N	-	-	In-house
E484K	-	E484K	-	-	Krammer lab
F486A	-	F486A	-	-	In-house
F486Y	-	F486Y	-	-	In-house
N487Q	-	N487Q	-	-	In-house
Y489F	-	Y489F	-	-	In-house
Q493A	-	Q493A	-	-	In-house
Q493N	-	Q493N	-	-	In-house
N501Y	-	N501Y	-	-	In-house
Y505A	-	Y505A	-	-	In-house
Y505F	-	Y505F	-	-	In-house
K417N/E484K/ L452R/N501Y	-	K417N/ E484K/ L452R/ N501Y	-	-	In-house
SARS-CoV-1 RBD WT					In-house
MERS-CoV RBD WT					In-house

Table S5. SARS-CoV-2 virus information and resource. Related to Figure 1 and 4.

Virus	S1 NTD	RBD	S1 CTD	S2	Source
D614G	-	-	D614G	-	2019-nCoV/USA-WA1/2020 D614G
B.1.351 Beta	L18F, D80A, D215G, L241del, L242del, A243del	K417N, E484K, N501Y	D614G	A701V	hCoV-19/USA/MD-HP01542/2021
P.1 Gamma	L18F, T20N, P26S, D138Y, G181V, R190S	K417T, E484K, N501Y	D614G, H655Y	T1027I, V1176F	hCoV-19/Japan/TY7-501/2021 from BEI
B.1.621 Mu	in3T, T95I, Y144S, Y145N,	R346K, E484K, N501Y	D614G, P681H	D950N	hCoV-19/USA/WI-UW-4340/2021
B.1.617.1 Kappa	G142D, E154K	L452R, E484Q	D614G, P681R	Q1071H, H1101D	hCoV-19/USA/CA-Stanford-15_S02/2021 from BEI
B.1.617.2 Delta	T19R, T95I, G142D, E156G, F157del, R158del	L452R, T478K	D614G, P681R	D950N	hCoV-19/USA/WI-UW-5250/2021
BA.1 Omicron	A67V, H69del, V70del, T95I, G142D, V143del, Y144del, Y145del, N211del, L212I, ins214EPE	G339D, S371L, S373P, S375F, K417N, N440K, G446S, S477N, T478K, E484A, Q493R, G496S, Q498R, N501Y, Y505H	T547K, D614G, H655Y, N679K, P681H	N764K, D796Y, N856K, Q954H, N969K, L981F	hCoV-19/USA/WI-WSLH-221686/2021
BA.2 Omicron	T19I, delL24, delP25, delP26, A27S, G142D, V213G	G339D, S371F, S373P, S375F, T376A, D405N, R408S, K417N, N440K, S477N, T478K,	D614G, H655Y, N679K, P681H	N764K, D796Y, Q954H, N969K	hCoV-19/Japan/UT-NCD1288-2N/2022

		E484A,<br>Q493R,<br>Q498R,<br>N501Y,<br>Y505H			
BA.2.75<br>Omicron	T19I,<br>delL24,<br>delP25,<br>delP26,<br>A27S,<br>G142D,<br>K147E,<br>W152R,<br>F157L,<br>I210V,<br>V213G,<br>G257S	G339H,<br>S371F,<br>S373P,<br>S375F,<br>T376A,<br>D405N,<br>R408S,<br>K417N,<br>N440K,<br>G446S,<br>N460K,<br>S477N,<br>T478K,<br>E484A,<br>Q498R,<br>N501Y,<br>Y505H	D614G,<br>H655Y,<br>N679K,<br>P681H	N764K,<br>D796Y,<br>Q954H,<br>N969K	hCoV-19/Japan/TY41-716/2022
BA.4<br>Omicron	T19I,<br>delL24,<br>delP25,<br>delP26,<br>A27S,<br>delH69,<br>delV70,<br>G142D,<br>V213G	G339D,<br>S371F,<br>S373P,<br>S375F,<br>T376A,<br>D405N,<br>R408S,<br>K417N,<br>N440K,<br>L452R,<br>S477N,<br>T478K,<br>F486V,<br>E484A,<br>Q498R,<br>N501Y,<br>Y505H,	D614G,<br>H655Y,<br>N679K,<br>P681H,	N764K,<br>D796Y,<br>Q954H,<br>N969K	hCoV-19/USA/MD-HP30386-PIDNBNVCCQ/2022
BA.5<br>Omicron	T19I,<br>delL24,<br>delP25,<br>delP26,<br>A27S,<br>delH69,<br>delV70,<br>G142D,<br>V213G	G339D,<br>S371F,<br>S373P,<br>S375F,<br>T376A,<br>D405N,<br>R408S,<br>K417N,<br>N440K,<br>L452R,<br>S477N,<br>T478K,<br>F486V,<br>E484A,<br>Q498R,<br>N501Y,	D614G,<br>H655Y,<br>N679K,<br>P681H,	N764K,<br>D796Y,<br>Q954H,<br>N969K	SARS-CoV-2/human/USA/COR-22-063113/2022

		Y505H,			
BL.1 Omicron	T19I, delL24, delP25, delP26, A27S, G142D, K147E, W152R, F157L, I210V, V213G, G257S	G339H, R346T, S371F, S373P, S375F, T376A, D405N, R408S, K417N, N440K, G446S, N460K, S477N, T478K, E484A, Q498R, N501Y, Y505H	D574V, D614G, H655Y, N679K, P681H	N764K, D796Y, Q954H, N969K	SARS-CoV-2/human /USA/WI-UW-12980/2022
XBB Omicron	T19I, L24del, P25del, P26del, A27S, V83A, G142D, Y144del, H146Q, Q183E, V213G,	G339H, R346T, L368I, S371F, S373P, S375F, T376A, D405N, R408S, K417N, N440K, V445P, G446S, N460K, S477N, T478K, E484A, F486S, F490S, Q498R, N501Y, Y505H,	D614G, H655Y, N679K, P681H,	N764K, D796Y, Q954H, N969K	hCoV-19/Japan/TY41-795/2022

Table S6. Pairs of S728-1157 and spike-WT-6P-Mut7 residues within predicted hydrogen bonding distances. Calculated using EpitopeAnalyzer[63] using a cutoff distance of 3.4 Å. **Related to Figure 3 and Figure S4.**

#	RBD Residue [Atom]	Ab Residue [atom]	Antibody Region	Distance (Å)	RBD residue mutated in Omicron VOC	RBD residue conserved across all VOC's
1	T415 [OG]	S56 [OG1]	CDRH2	2.78	No	Yes
2	Y421 [OH]	S53 [O]	CDRH2	2.73	No	Yes
3	Y453 [OH]	D98 [OD1]	CDRH3	3.5	No	Yes
4	L455 [O]	Y33 [OH]	CDRH1	3.29	No	Yes
5	R457 [O]	S53 [OG]	CDRH2	3.25	No	Yes
6	Y473 [OH]	R31 [O]	CDRH1	2.76	No	Yes
7	Y473 [OH]	S53 [OG]	CDRH2	3.26	No	Yes
8	Q474 [O]	R31 [NH1]	CDRH1	3.08	No	Yes
9	A475 [O]	L28 [N]	CDRH1	3.05	No	Yes
10	A475 [O]	N32 [ND2]	CDRH1	2.98	No	Yes
11	E484 [OE2]	Y99 [OH]	CDRH3	2.61	Yes	No
12	N487 [ND2]	G26 [O]	FR1	3.01	No	Yes
13	C488 [O]	Y99 [OH]	CDRH3	3.25	No	Yes
14	Y489 [OH]	R94 [NH1]	FR3	2.64	No	Yes
15	Y505 [OH]	Q31 [NE2]	CDRL1	2.62	Yes	No

Table S7. Buried surface area (BSA) of antibody interface residues by SARS-CoV-2 RBD. *indicates van der Waals interactions other than polar or hydrophobic interactions. BSA is calculated using PISA program(79). Somatically mutated residues are underlined. **Related to Figure 3, Figure S4** and **Table S6.**

Antibody	Residue #	Hydrogen bond	BSA (Å^2)
S728-1157 heavy chain	GLU 1		42.9
	VAL 2		11.7
	GLY 26	Yes	41.7
	LEU 27	*	10.5
	LEU 28	Yes	36.9
	SER 30		5.8
	ARG 31	Yes	106.7
	ASN 32	Yes	11.8
	TYR 33	Yes	74.4
	TYR 52	*	53.9
	SER 53	Yes	43.7
	GLY 54	Yes	39.7
	GLY 55		21.9
	SER 56	Yes	31.5
	THR 57		1.6
	PHE 58	*	40.7
	ARG 94	Yes	39.1
	ASP 95		0.2
	LEU 96		39.0
	SER 97		26.9
	ASP 98	Yes	55.0
	TYR 99	Yes	123.7
	ASP 101		14.3

S728-1157 light chain	ASP 26		4.2
	ASN 27		4.1
	VAL 28	*	51.5
	SER 30		44.3
	GLN 31	Yes	31.9
	ASN 32		10.3
	ARG 50		30.9
	GLY 68		7.6
	TRP 91	*	46.8
	ASP 92		13.2
	SER 93		22.8
	SER 94		15.4

Antibody	Residue #	Hydrogen bond	BSA (Å^2)
C12.3 heavy chain	GLN 1		12.7
	VAL 2		11.9
	GLY 26	Yes	18.2
	PHE 27	*	17.7
	THR 28	Yes	30.4
	SER 30		4.2
	SER 31	Yes	67.8
	ASN 32	Yes	23.6
	TYR 33	Yes	54.7
	TYR 52	*	47.6
	SER 53	Yes	44.1
	GLY 54	Yes	62.2
	GLY 55		0.6
	SER 56	Yes	48.5
	THR 57		1.0
	PHE 58	*	41.2
	ARG 71		0.7
	ARG 94	Yes	43.9
	ASP 95		0.6
	PHE 96		74.1
	GLY 97	Yes	23.0
	ASP 98		9.6
	PHE 99	*	61.7
	ASP 101		0.6
	TYR 102	*	19.8
C12.3 light chain	ILE 2		0.8
	SER 28	Yes	31.1
	VAL 29		4.7
	SER 30		33.6
	TYR 32	Yes	41.9
	TYR 91		0.4
	GLY 92	Yes	37.9
	SER 93	Yes	13.8

Table S8. Cryo-EM data collection, refinement and model building statistics. Related to Figure 3 and Figure S4.

**Map**	**S728-1157 + SARS-CoV-2-6P-Mut7 (global refinement)**	**S728-1157 + SARS-CoV-2-6P-Mut7 (focused refinement)**
EMDB	EMD-27112	EMD-27113
**Data collection**		
Microscope	Thermo Fisher Titan Krios	
Voltage (kV)	300	
Detector	Gatan K2 Summit	
Recording mode	Counting	
Nominal magnification	130kx	
Movie micrograph pixelsize (Å)	1.045	
Dose rate (e^-/[(camera pixel)*s])	6.017	
Number of frames per movie micrograph	36	
Frame exposure time (ms)	250	
Movie micrograph exposure time (s)	9	
Total dose (e^-/Å^2)	50.0	
Defocus range (μm)	-0.8 to -1.5	
**EM data processing**		
Number of movie micrographs	1,718	1,718
Number of molecular projection images in map	151,948	29,595
Symmetry	C1	C1
Map resolution (FSC 0.143; Å)	3.3	3.7
Map sharpening B-factor (Å^2)	-85.3	-71.1
**Structure Building and Validation**		
Number of atoms in deposited model		
SARS-CoV-2-6P-Mut7	*n/a*	20,759
Fab Fv	*n/a*	1,653
Glycans	*n/a*	182
MolProbity score	*n/a*	1.07
Clashscore	*n/a*	1.66
Map correlation coefficient	*n/a*	0.75
EMRinger score	*n/a*	2.57
d FSC model (0.5; Å)	*n/a*	3.8
RMSD from ideal		
Bond length (Å)	*n/a*	0.021
Bond angles (˚)	*n/a*	1.81
Ramachandran plot		
Favored (%)	*n/a*	97.13
Allowed (%)	*n/a*	2.87
Outliers (%)	*n/a*	0.00
Side chain rotamer outliers (%)	*n/a*	0.08
Cβ outliers (%)	*n/a*	0.00
PDB	*n/a*	8d0z
